# Supplementary figures and images for: Extended receptor repertoire of an adenovirus associated with human obesity
Source: PLoS Pathog. 2025 Jan 30;21(1):e1012892. doi: 10.1371/journal.ppat.1012892 (PMC11813153; doi:10.1371/journal.ppat.1012892)

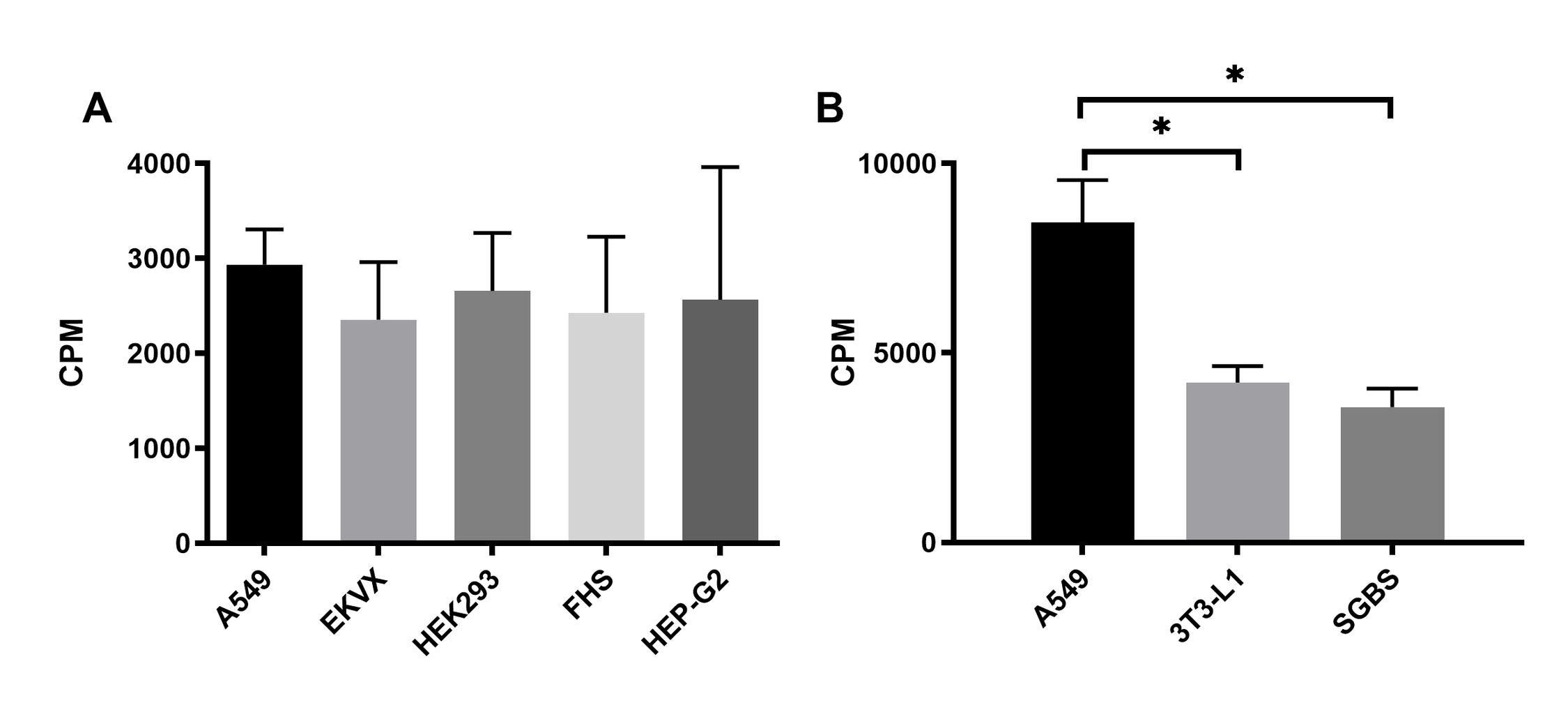

Supplement: S1 Fig — A Binding of 35S-labelled HAdV-D36 virions to different human-derived cell lines A549 (adenocarcinomic human alveolar basal epithelial cells), EKVX (human lung adenocarcinoma cell line), HEK293 (human embryonic kidney 293 cells), FHS (human small intestine cell line), HEP-G2 (human hepatocellular carcinoma cell line). B Binding of 35S-labelled HAdV-D36 virions to A549, 3T3-L1 (mouse fibroblasts that can differentiate into an adipocyte-like phenotype) and SGBS (human Simpson-Golabi-Behmel syndrome preadipocyte cell line) cells. The amount of virus particles bound to cells was presented as counts per minute (CPM). All experiments were performed three times with duplicate samples. Error bars represent mean ± SD. The statistical significance was determined by unpaired two-tailed t test; * P of < 0.05. (TIF) [file ppat.1012892.s001.tif]

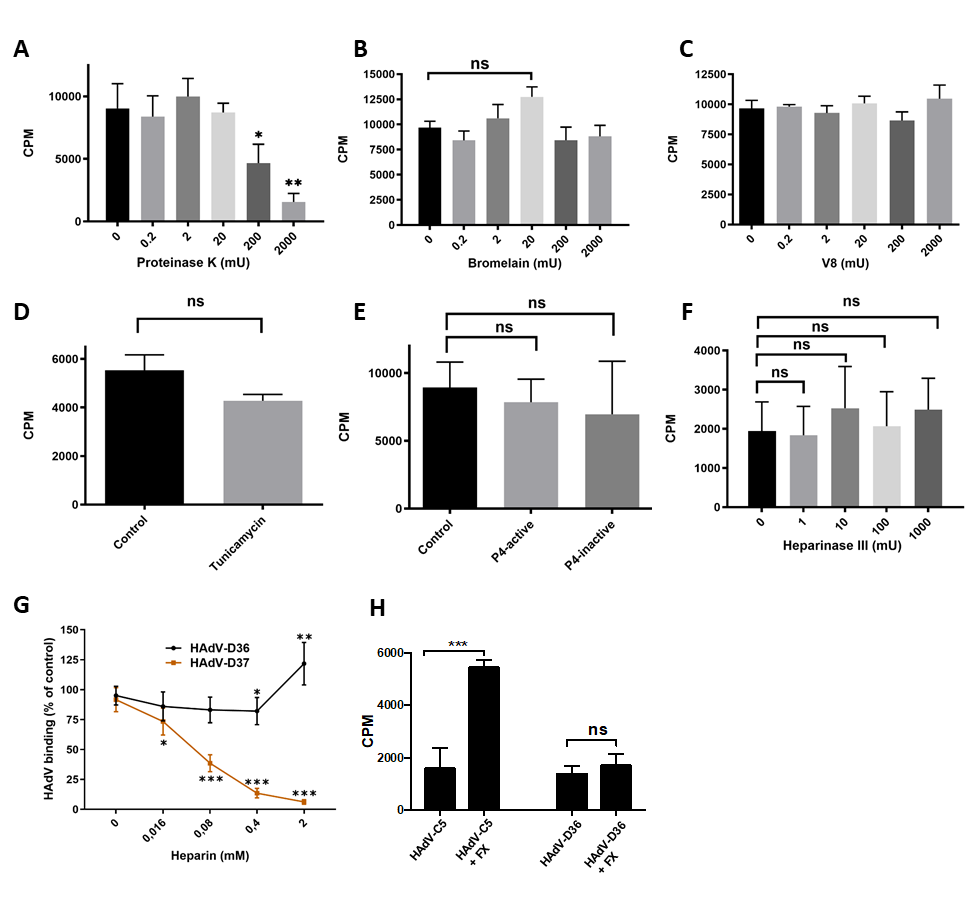

Supplement: S2 Fig — Binding of 35S-labelled HAdV-D36 virions to A549 cells treated with different proteases. A Proteinase K (a broad-spectrum serine-protease), B Bromelain (a mixture of pineapple cysteine proteases), C V8 (an endo-proteinase Glu-C serine protease cleaving after glutamic acid), D tunicamycin (an inhibitor of N-glycosylation), E, P4 (DL-threo-1-phenyl-2-palmitoylamino-3-pyrrolidino-1-propanol, an inhibitor of glycolipid synthesis) and F heparinase III (10 mU/μL, which cleaves heparan sulfate from cell surface). G Attachment inhibition assay with soluble heparin (Mw approx. 21000). HAdV-D37 was used as a positive control. H 35S-labeled HAdV-D36 and HAdV-C5 virions, pre-incubated with physiological concentrations of coagulation factor X (FX: 10 μg/mL), binding to A549 cells. In figure A-F and H, y-axis shows the amount of virus particles bound to cells and represented as CPM (count per minute). All experiments were performed three times with duplicate samples. Error bars are representing mean ± SD. The statistical significance was determined by unpaired two-tailed t test; ns = not significant, * P of < 0.05, ** P of < 0.01 and *** P of < 0.001. (TIF) [file ppat.1012892.s002.tif]

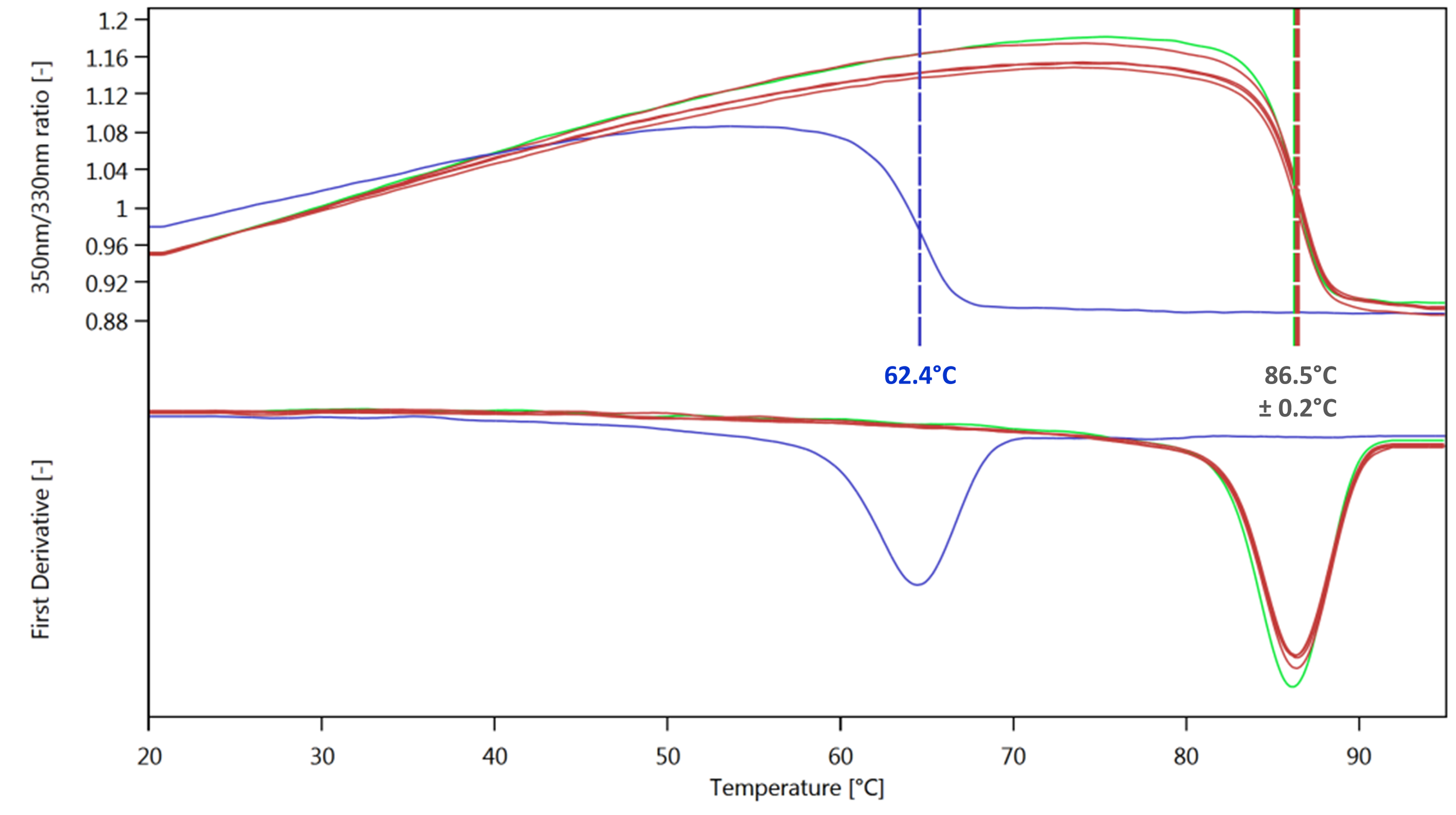

Supplement: S3 Fig — The scan was performed using a Prometheus NT.48. The ratio of the fluorescence at 350 nm and 330 nm is plotted against the temperature (upper part). The first derivative calculation can be deduced to determine the Tm (lower part). At pH 7.5 (red and green curves, n = 5), a Tm of 86.3–86.5°C was observed. At a pH of 2–3 (blue), the FK stability was lowered, but still high (Tm = 62.4°C). The experiment was performed in the course of a Prometheus demonstration by Dr. Fabian Zehender (NanoTemper Technologies GmbH). (TIF) [file ppat.1012892.s003.tif]

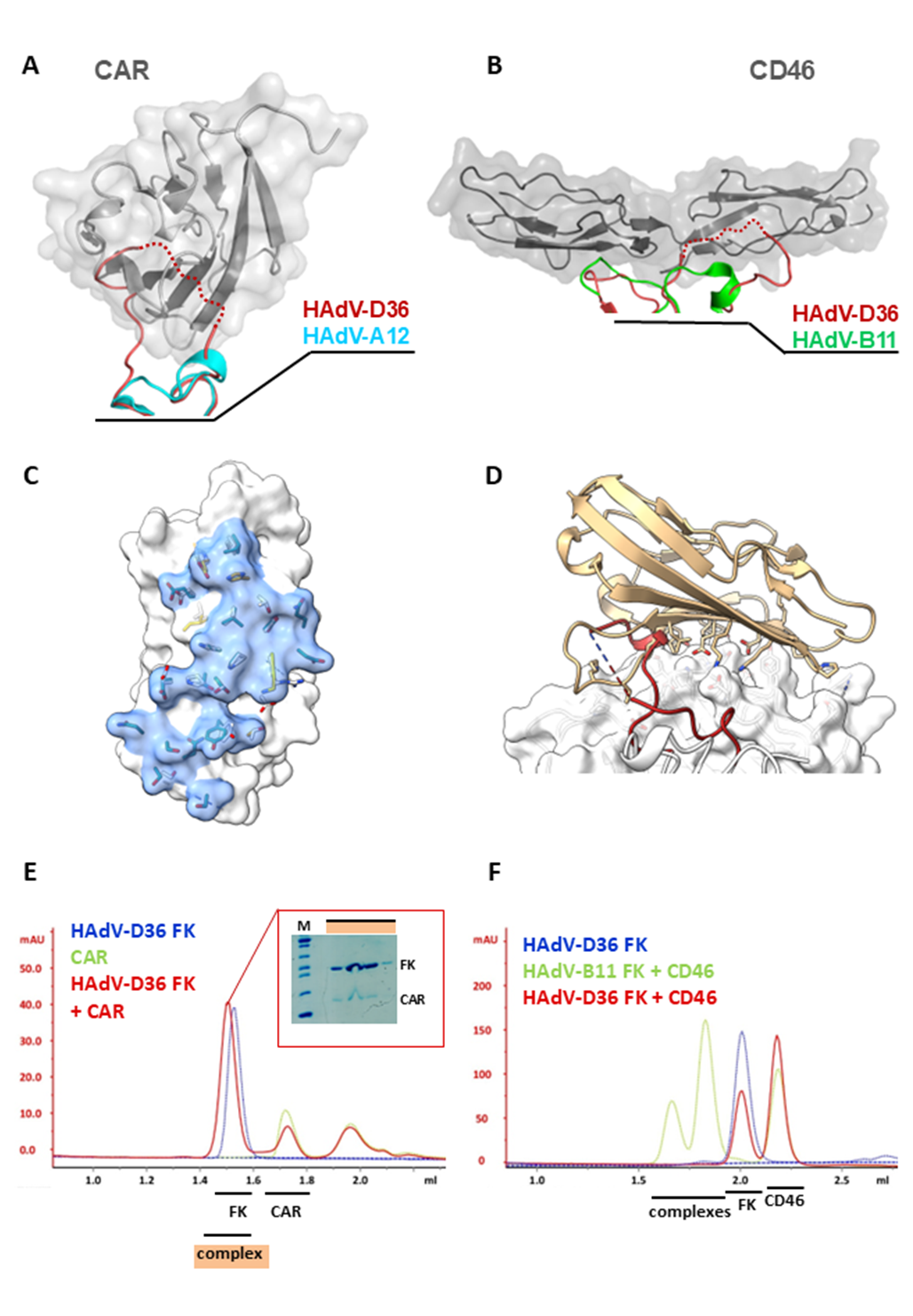

Supplement: S4 Fig — A Superposition of HAdV-D36 FK (red) onto the HAdV-A12 FK (blue) in the CAR (light gray) complex structure (PDB-ID 1KAC). The HAdV-D36 FK heavily clashes with CAR through its DG loop (red dotted line). B Superposition of HAdV-D36 FK (red) onto HAdV-B11 (green) in the CD46 complex structure (PDB-ID 2O39). The HAdV-D36 DG loop is located proximally in front of CD46 (dark gray) and would likely interfere with CD46 binding without producing direct clashes. Superpositions of fiber knobs and the corresponding complex structures were performed using the ‘align’ algorithm in PyMol. C Conservation of the CAR-interface between HADV-D36 and HAdV-D37. The surface of HAdV-D37 FK (PDB 2J12) is shown in surface representation, and the CAR interface is colored blue. The residues of HAdV-D37 that form the interface are shown in teal. HAdV-D36 FK was superposed using the monomers, and its residues are shown in white. Residues that are only functionally conserved are colored yellow, residues that are not conserved are colored orange. D The canonical interface between HAdV-D36 FK and CAR is disturbed by the DG loop. The structure of HAdV-D36 was superposed on the HAdV-D37 FK CAR complex (PDB 2J12), superposing the monomers. HAdV-D36 is shown in white, CAR in gold. Although the resulting interface is highly shape complementary, the HAdV-D36 DG loop from the adjacent chain (red) is disturbing the interaction. E SEC of the HAdV-D36 FK / CAR complex (red line). HAdV-D36 FK (blue line) was incubated with soluble CAR-D1 (green line) for 20 minutes prior to SEC. The peak fractions (orange bar) of SEC were characterized by SDS-PAGE shown in inset. Equal amounts of FK and CAR were used for optimal comparability. F Comparison of SEC profile of complex formation of HAdV-D36 (blue line) with CD46 (green line) to HAdV-B11 with CD46. The elution profile of HAdV-D36 FK-CD46 is shown with red line while the green line depicts that of HAdV-D36 FK-CD46. The proteins causing the respective peaks are note [file ppat.1012892.s004.tif]

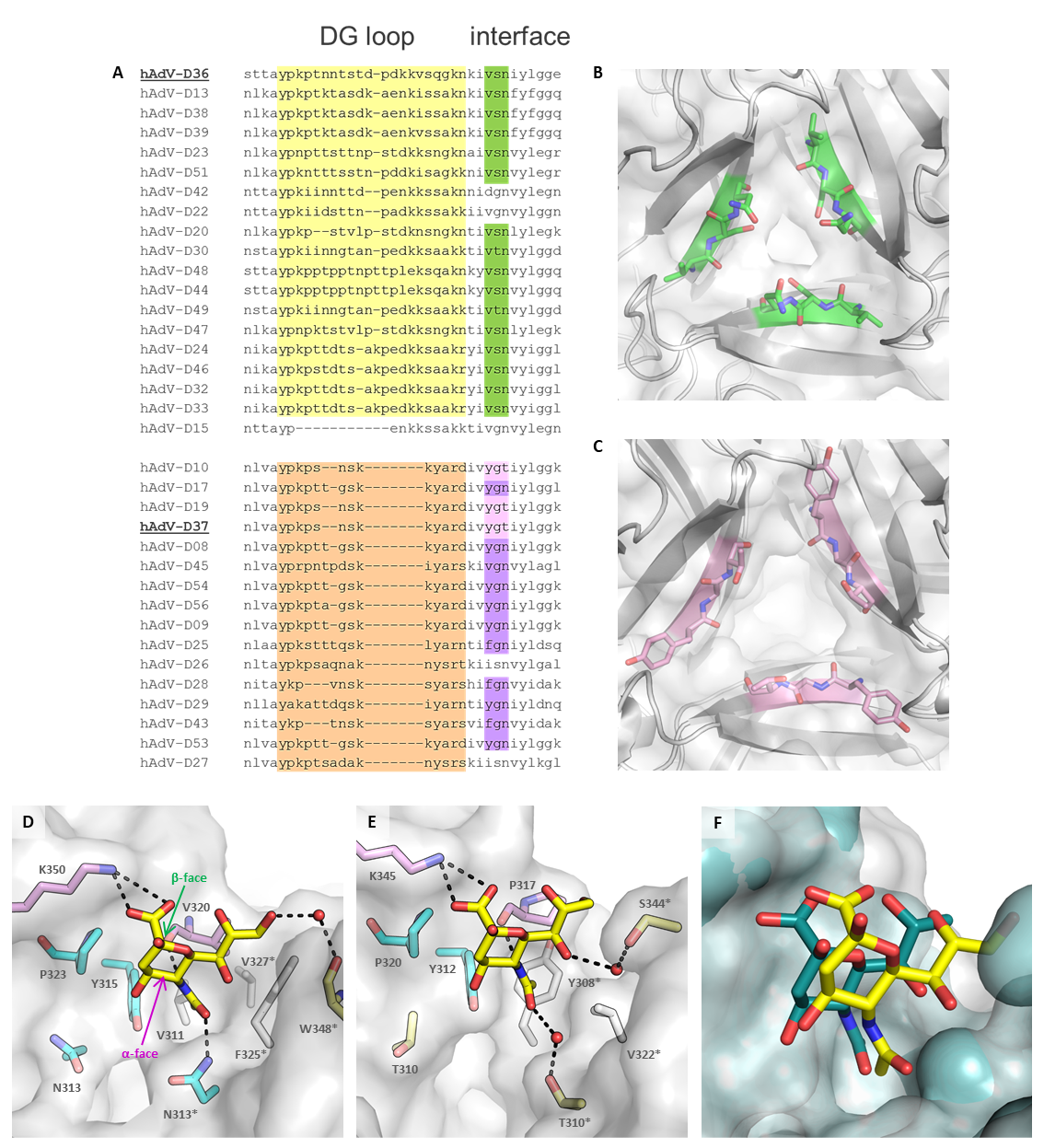

Supplement: S5 Fig — A Excerpt of the alignment of all species D HAdVs from type HAdV-D08 to D56 performed with Clustal Omega [116]. The 18 HAdV types belonging to the ‘HAdV-D36-like’ clade possess elongated DG loops (yellow) and a VSN or VTN interface configuration (green). The 17 types belonging to the ‘HAdV-D37-like’ clade have shorter DG loops (orange) and a YGT (light pink) or YGN (purple) interface configuration. B The VSN trimer interface (green) as found in the HAdV-D36 FK. C YGT trimer interfaces (light pink) as observed in the HAdV-D37 FK (PDB-ID 1UXE). D Neu5Ac (yellow) binding mode observed for the HAdV-D36 FK (gray surface). The structure was solved with α-2-O-methyl-Neu5Ac, and methyl functions have been left out for clarity. Residues marked with an asterisk are contributed by the clockwise neighboring monomer. Key contacts are mediated by K350 and V320 (purple), while the hydrophobic binding cavity for the N-acetyl function is formed by V311, V327*, and F352*. W348 (yellow) contacts the glycerol function by a water-bridged contact. The three residues N313, Y315, and P323 (cyan) put steric constraints onto the sugar’s O4 atom, while N313* contacts the N-acetyl group by means of a hydrogen bond from the α-face. The α- and β- faces of Neu5Ac are marked with an arrow. E Neu5Ac binding mode observed for HAdV-D37 (PDB-ID 1UXA). Coloring according to A. Unlike N313 in HAdV-D36, T310 (yellow) does not put steric pressure onto the Neu5Ac O4, and T310* contacts the sugar via a water-bridged polar contact. F Relative placement of Neu5Ac complexed to HAdV-D36 FK (yellow) and HAdV-D37 FK (teal) upon superposition of the knob trimers. The sugars show a prominent relative shift. (TIF) [file ppat.1012892.s005.tif]

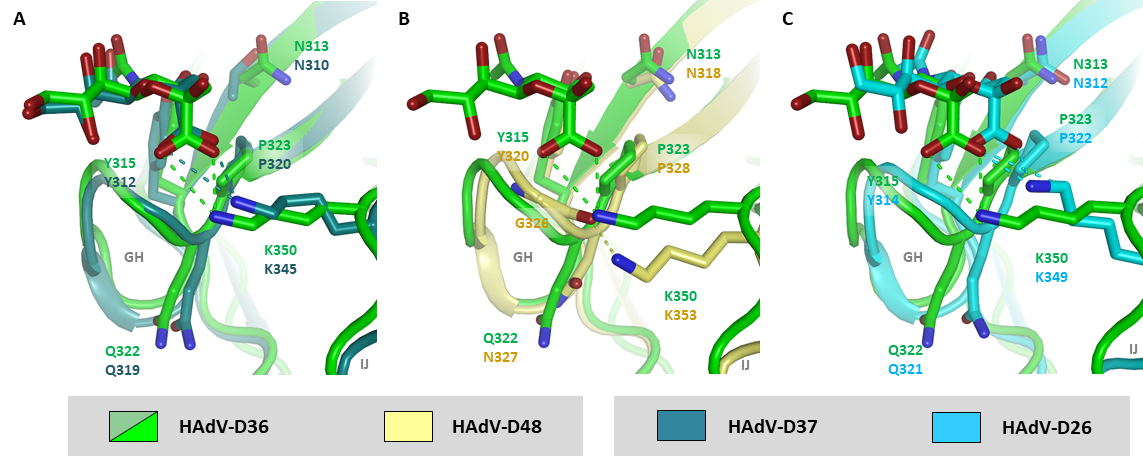

Supplement: S6 Fig — A Comparison of SA binding of HAdV-D36 with HAdV-D37 upon superposition of only one monomer. B Comparison of SA binding of HAdV-D36 with HAdV-D48 upon superposition of only one monomer. C Comparison of SA binding of HAdV-D36 with HAdV-D26 upon superposition of only one monomer. Only the displayed chain was used for superpositioning the structures in PyMol. (TIF) [file ppat.1012892.s006.tif]

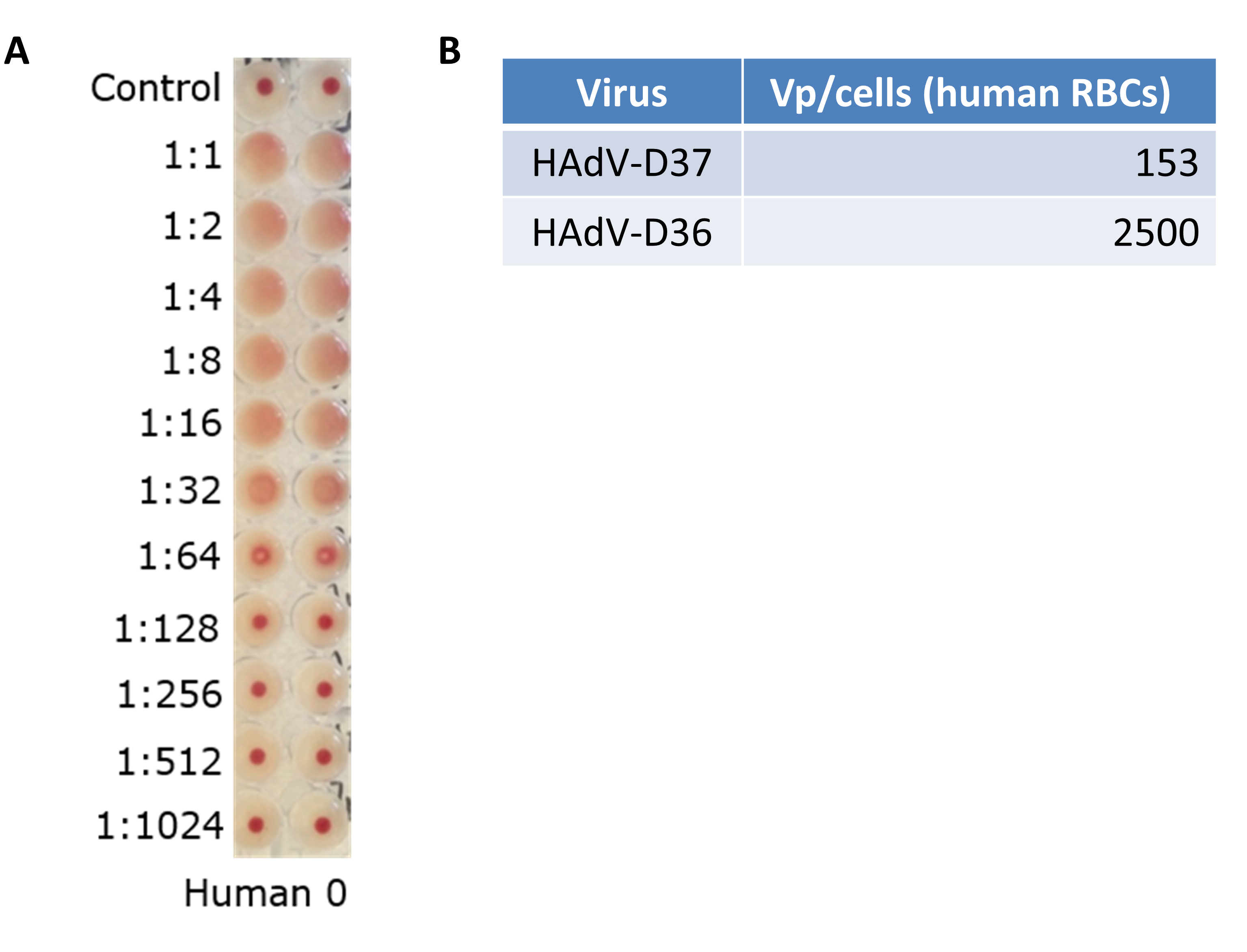

Supplement: S7 Fig — A Depiction of the hemagglutination experiment. Human blood was diluted as indicated in the methods. B Quantification of the number of virus particles per cell required for complete agglutination of human red blood cells. (TIF) [file ppat.1012892.s007.tif]

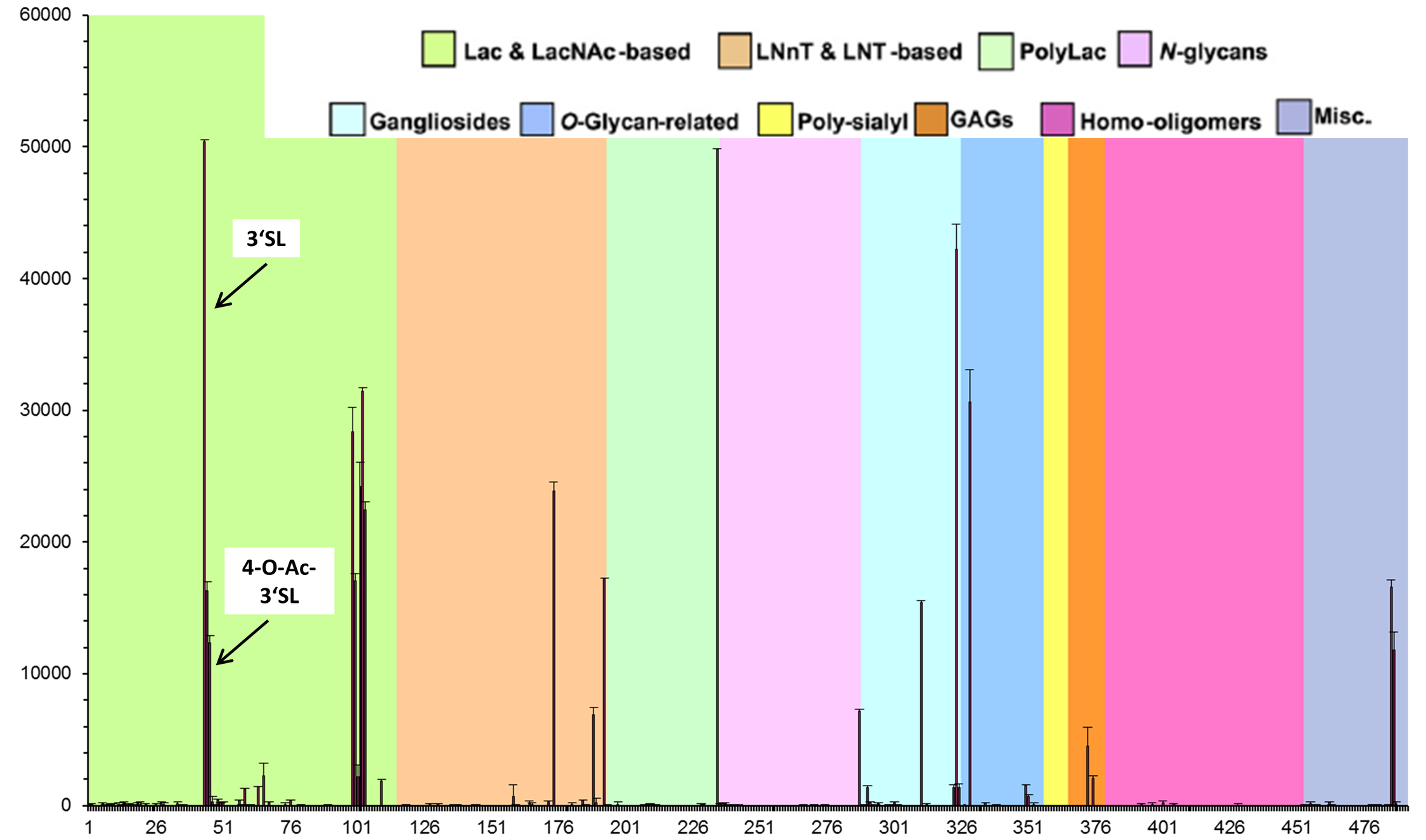

Supplement: S8 Fig — A large glycan array containing 498 glycans (see legend on the upper right) was in good overall agreement with previous findings [36]. Numerical scores for the binding intensity are shown as means of fluorescence intensities of duplicate spots at 5 fmol/spot, with error bars representing half of the difference between the two values. (TIF) [file ppat.1012892.s008.tif]

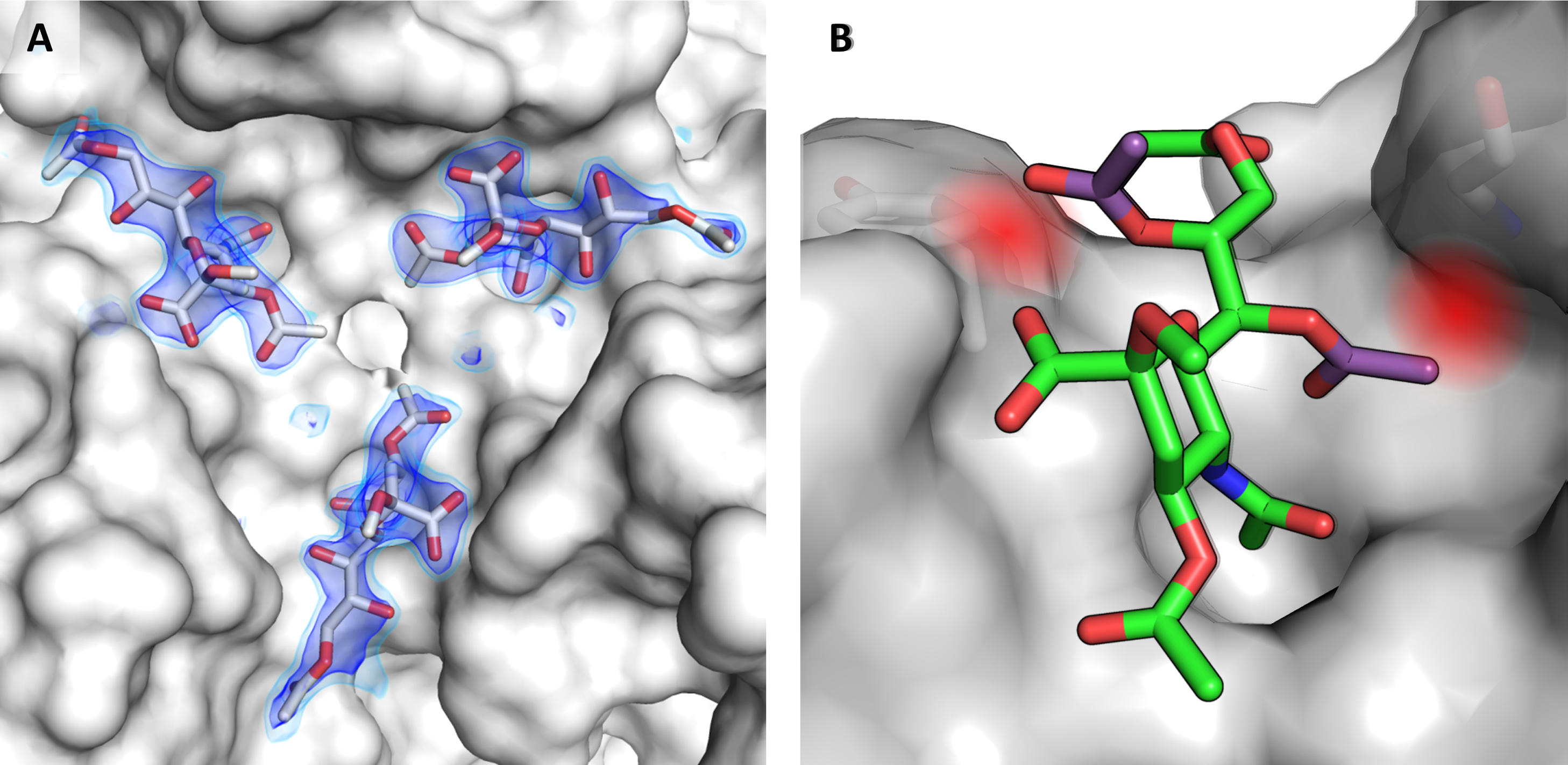

Supplement: S9 Fig — A Complex structure of HAdV-D36 with synthetic α-2-O-methyl-Neu4,5,9Ac3. Shown is a 2Fo-Fc map contoured at 1.5σ (dark blue) and 1σ (light blue), respectively. The additional 9-O-acetyl group displays various conformations and does not contribute to the binding. B Modelling of additional O-acetylations at position 7 and 8 (purple). Modelling was performed in Coot without application of a force field. In agreement with [71], the acetyl groups were added in a sterically favorable position that retains good ligand geometry. Steric clashes are probable in both cases (red discs represent the clashing regions). (TIF) [file ppat.1012892.s009.tif]

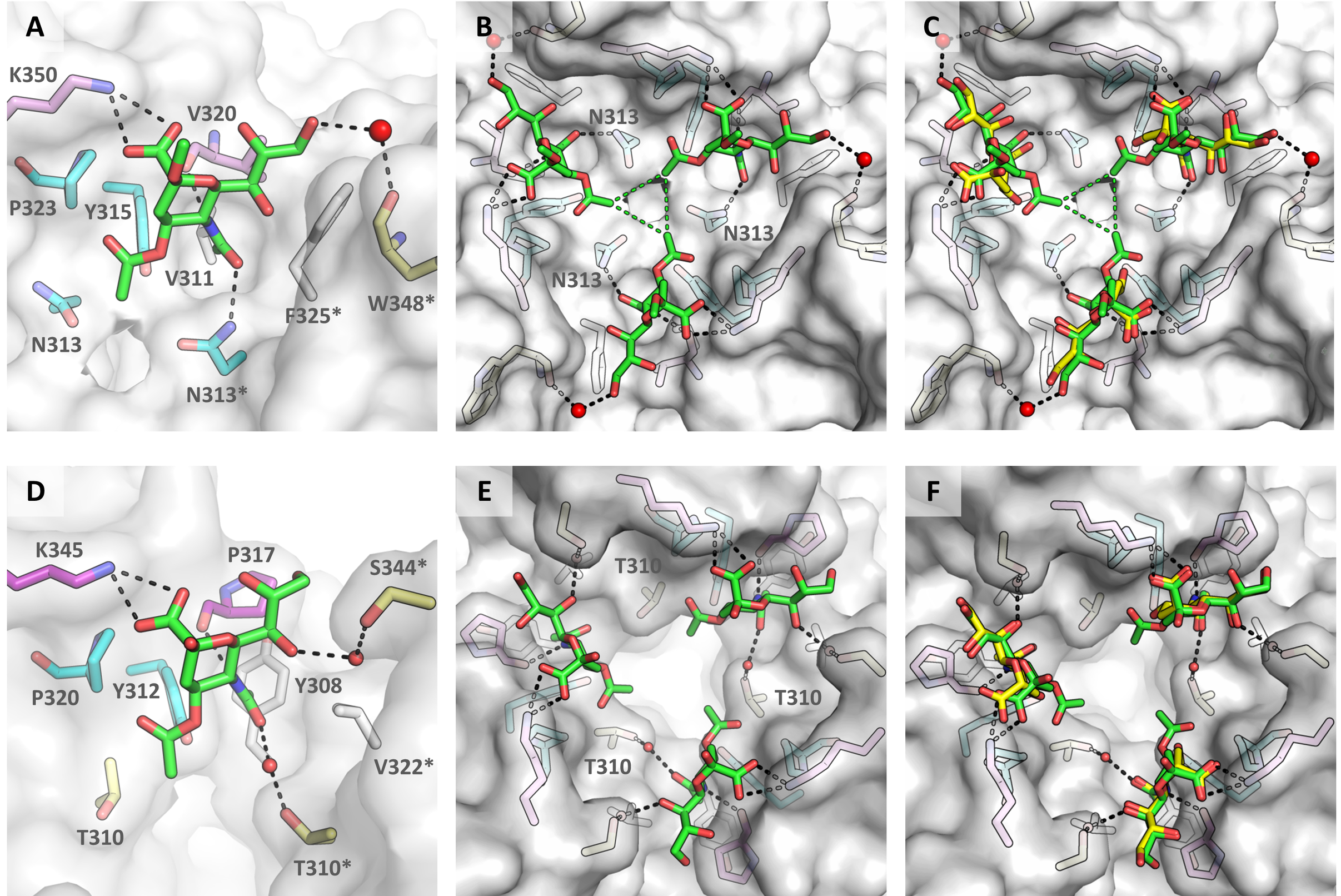

Supplement: S10 Fig — Coloring of interacting residues according to Fig 5. A-C Neu4,5Ac2 (green) binding mode observed for the HAdV-D36 FK (gray surface) analogous to Fig 5A–5B. D-F Neu4,5Ac2 (green) binding mode observed for the HAdV-D37 FK (gray surface). The triangular hydrophobic interaction is not observed in HAdV-D37. Instead, Neu4,5Ac2 moieties are located in the periphery of the binding cavity. The relative movement between Neu5Ac (yellow, PDB-ID 1UXA) and Neu4, 5Ac2 is less pronounced for HAdV-D37 than for HAdV-D36. (TIF) [file ppat.1012892.s010.tif]

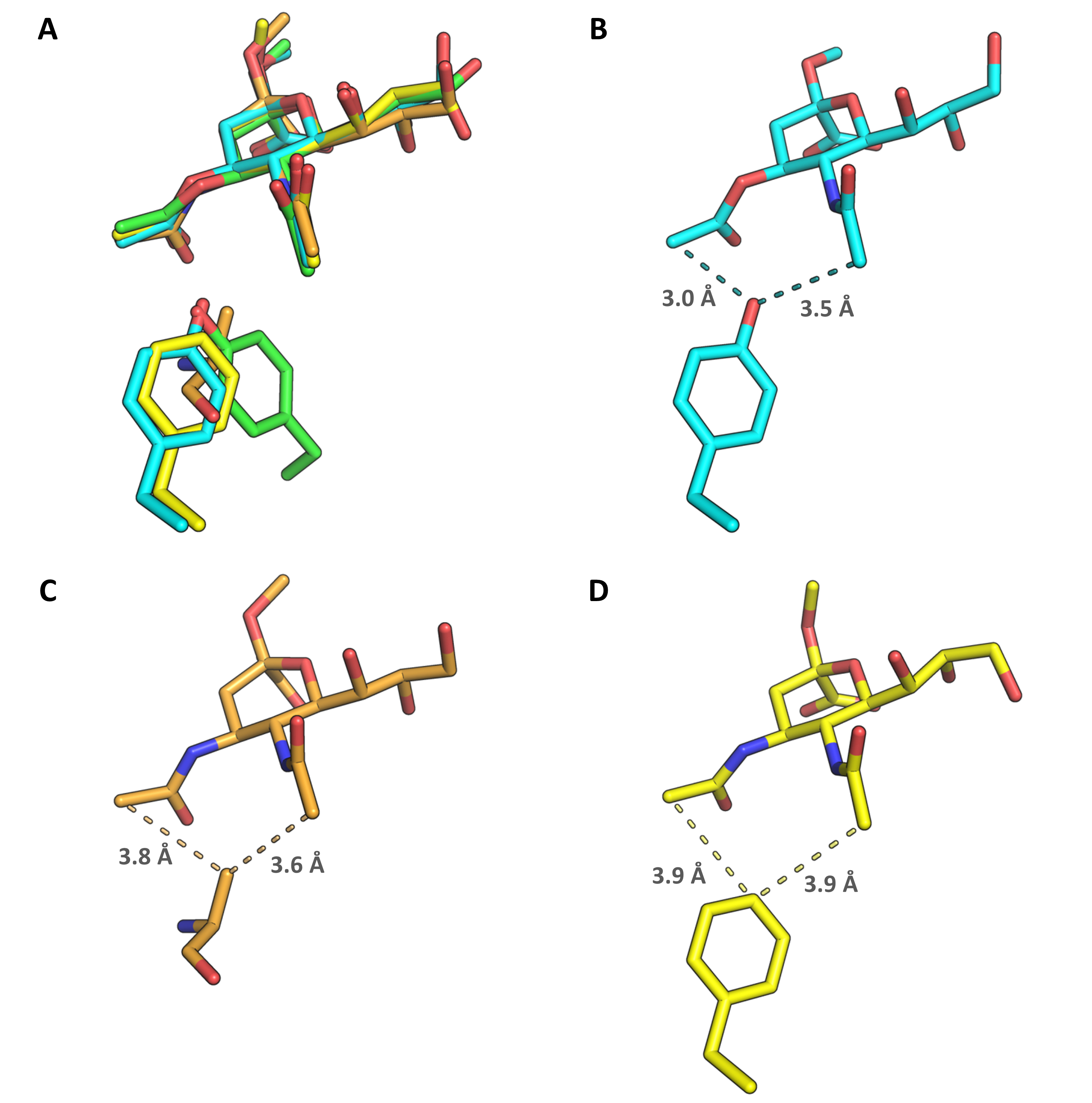

Supplement: S11 Fig — Superpositioning was done according to the carbohydrate portions using PyMol. A Superposition of HAdV-D36 (green), mouse hepatitis virus strain S hemagglutinin-esterase (MHV-S HE0, cyan, PDB ID 4C7W), and the lectin (yellow) and catalytic (orange) sites of rat coronavirus strain New-Jersey hemagglutinin-esterase (RCoV-NJ HE0, PDB ID 5JIL) in complex with the non-hydrolysable Neu4,5Ac2 analogue α-2-O-methyl-Neu4,5-di-N-acetylneuraminic acid. B Gatekeeping residue of MHV-S HE0. C, D Gatekeeping residues of the RCoV-NJ HE0 esterase (orange) and lectin (yellow) binding sites. (TIF) [file ppat.1012892.s011.tif]

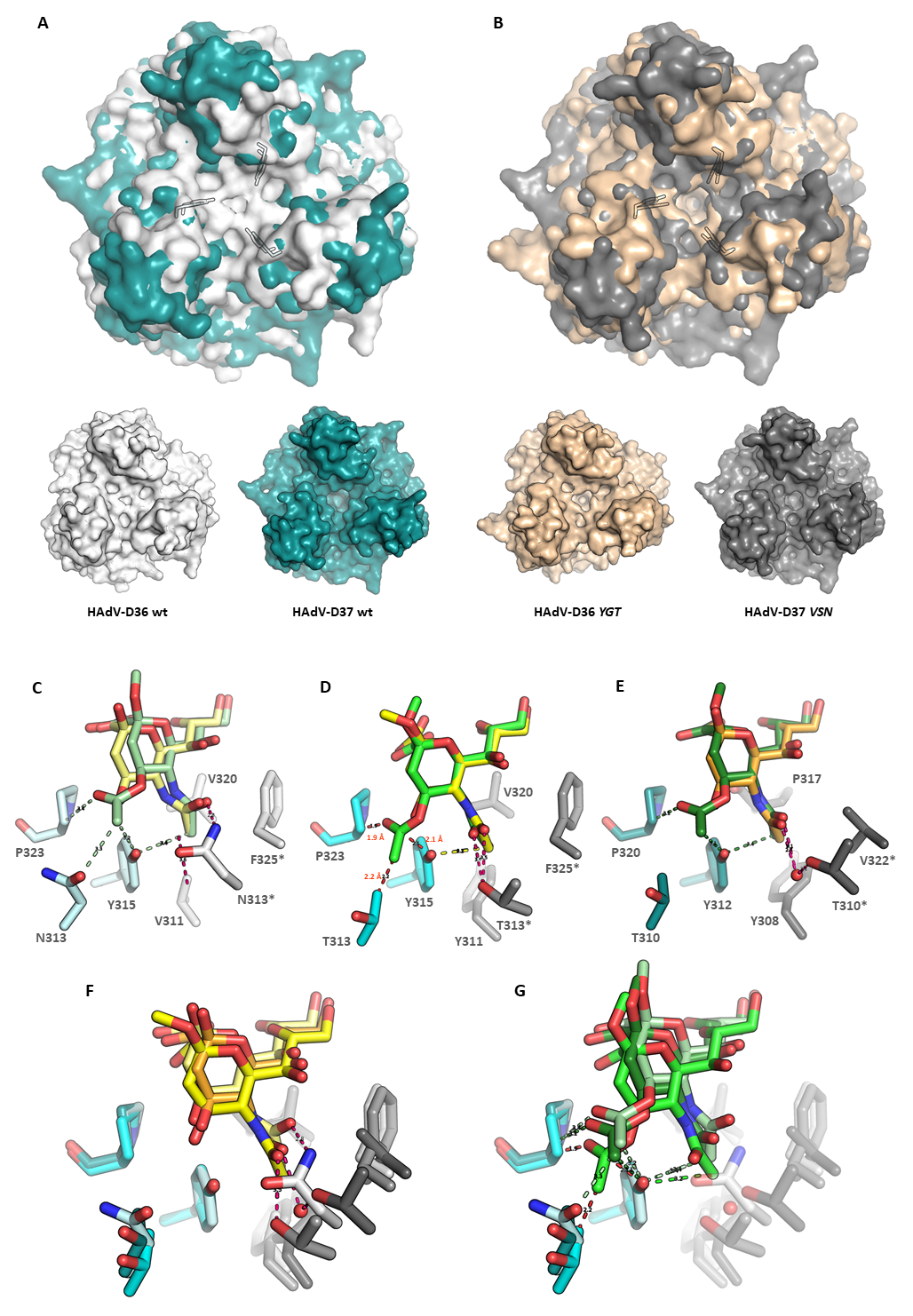

Supplement: S12 Fig — All superpositions were performed in PyMol aligning all atoms of the trimer. A Superposition of the HAdV-D36 wt FK (gray) with the HAdV-D37 wt FK (teal) in analogy to Fig 3B. Below are the separate surface representations of the two wt knobs. B Superposition of the HAdV-D36 YGT FK (orange) with the HAdV-D37 VSN FK (dark gray). Below are the separate surface representations of the two mutant knobs. The HAdV-D37 VSN FK shows a narrower central cavity than HAdV-D36 YGT. A clockwise movement of the Y317 side chain in HAdV-D36 YGT distorts the N-acetyl binding cavity (both analogous tyrosine residues shown as sticks). C Superposition of SA complexes of HAdV-D36 wt. The interacting residues were aligned on the sidechain of the gatekeeping residue Y315. The O-acetyl group of Neu4,5Ac2 (green) is in the van-der-Waals range of sidechains N313, Y315, and P323, while the N-acetyl group forms a direct hydrogen bond with N313*. D Modeling of Neu4,5Ac2 (green) on the HAdV-D36/Neu5Ac complex structure. Neu4,5Ac2 was aligned onto Neu5Ac (yellow) in Coot. The relative spacing of T313* and the N-acetyl group prevent the formation of a water-mediated contact. Instead, the N-acetyl group moves towards T313* and forms a direct, long hydrogen bond. Thereby, the N-acetyl function is slightly rotated out of its ideal position and causes a rotation of the whole ligand. As a consequence, the O-acetyl function of a possible Neu4,5Ac2 complex would produce heavy clashes with T313, Y315, and P323 that cannot be overcome by a rotation towards the center. E Superposition of SA complexes of HAdV-D37 wt. T310* is located further away from the sugar’s N-acetyl group and allows for the formation of a water-mediated contact in both cases. F Superposition of the Neu5Ac complex structures of HAdV-D36 wt, HAdV-D36 YGT, and HAdV-D37 wt. The need to form a direct contact in HAdV-D36 YGT pulls the sugar towards the gatekeeping residue Y315. Coloring according to C-E. G Superposition of the Neu4,5Ac2 complex [file ppat.1012892.s012.tif]

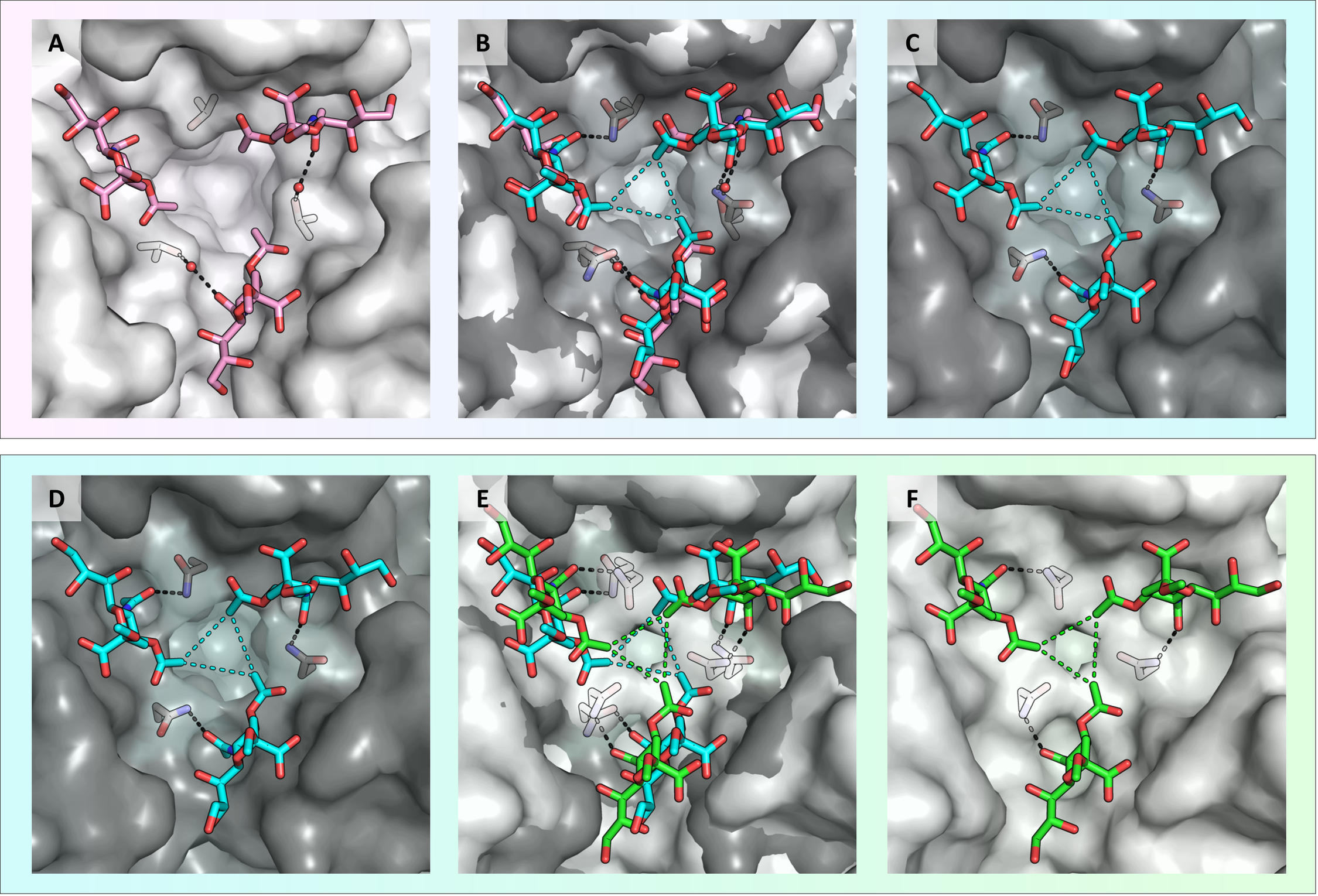

Supplement: S13 Fig — Residues at the analogous positions 310 (HAdV-D37) and 313 (HAdV-D36) are displayed as sticks and their contacts with the sugar as black dashed lines. A Binding mode of HAdV-D37 wt (pink sticks, light surface). B Superposition of the Neu4,5Ac2 complex structured of HAdV-D37 wt and VSN (cyan sticks, dark surface). The triangular hydrophobic contact is restored in the VSN mutant. C, D Binding mode of HAdV-D37 VSN. The same panel is displayed twice for reasons of clarity. E Superposition of the Neu4,5Ac2 complex structures of HAdV-D37 VSN and HAdV-D36 wt (green sticks, light surface). Despite a relative clockwise and upward positioning of the sugars in HAdV-D36, the length of the triangular hydrophobic contact is very similar. F Binding mode of HAdV-D36 wt analogous to Fig 5D. (TIF) [file ppat.1012892.s013.tif]

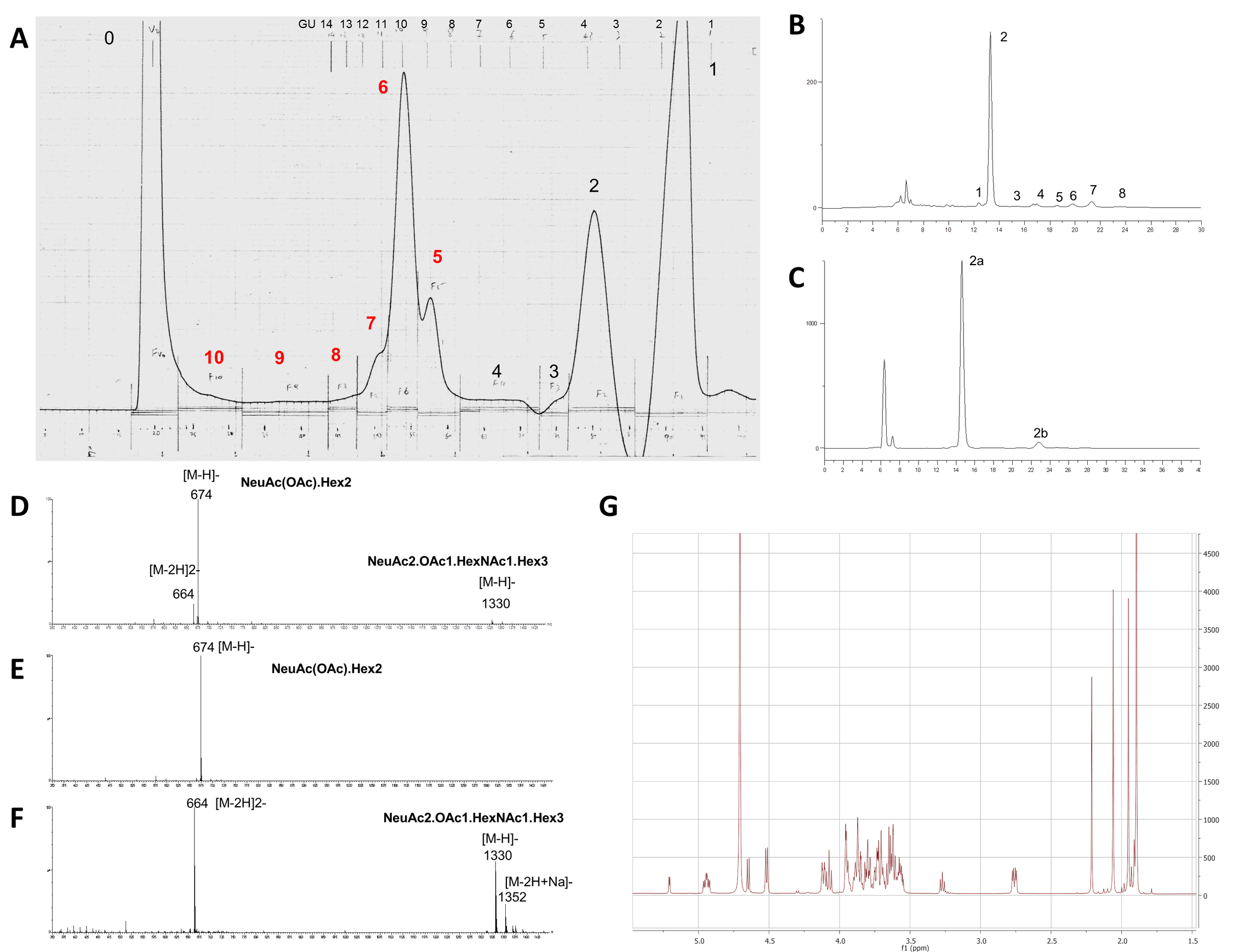

Supplement: S14 Fig — A Bio-Gel P4 Fractionation of EMOs. B HPLC of Bio-Gel P4 fraction F6 C Re-HPLC of Bio-Gel P4 fraction F6-2. D Negative-ion mass spectra of HPLC fraction F6-2. E Negative-ion mass spectra of HPLC fraction F6-2a F Negative-ion mass spectra of HPLC fraction F6-2b. G 1H-NMR of HPLC F6-2a. (TIF) [file ppat.1012892.s014.tif]
