# Supplementary material for: Extended receptor repertoire of an adenovirus associated with human obesity
Source: PLoS Pathog. 2025 Jan 30;21(1):e1012892. doi: 10.1371/journal.ppat.1012892 (PMC11813153; doi:10.1371/journal.ppat.1012892)
Supplement: S1 Table — (PDF) [file ppat.1012892.s015.pdf]

Supporting information

S1 Table. List of probes included in microarray screening analysis (Array Sets 32-39); their sequences and fluorescence intensities at 5 fmol per spot of binding with His-tagged fiber knobs of HAdV-D36 and HAdV-D37

Position	Probe	Structure	HAdV-D36	HAdV-D37
1	Galactocerebrosides	Galβ-Cer	-	85
2	H-Di	Fucα-2Gal-DH	-	40
3	A-Tri	GalNAcα-3Gal-DH<br>| Fucα-2	-	-
4	B-Tri	Galα-3Gal-DH<br>| Fucα-2	143	-
5	B-Tri-AO	Galα-3Gal-AO<br>| Fucα-2	-	-
6	GSC-426	(3-deoxy,3-carboxymethyl)Galβ-C30	182	118
7	Sulfatide	SU-3Galβ-Cer	-	-
8	GSF-1	SU-3Galβ-C30	-	-
9	GSC-209	GlcAβ-3Galβ-Cer42	-	61
10	GSC-210	SU-3GlcAβ-3Galβ-Cer42	140	98
11	GSC-187	NeuAcα-3Galβ-C29	-	-
12	GSC-40	NeuAcα-(S)-3Galβ-Cer42	-	93
13	GSC-230	NeuAcα-8NeuAcα-3Galβ-Cer36	-	-
14	GSC-27	NeuAcα-6Galβ-Cer36	-	188
15	GSC-144	KDNα-6Galβ-Cer36	-	131
16	GSC-13	NeuAcα-(S)-6Galβ-Cer36	-	2
17	GSC-72	NeuAcα-(S)-6Galβ-(S)-Cer36	-	13
18	GSC-231	NeuAcα-8NeuAcα-6Galβ-Cer36	-	-
19	GSC-439	NeuAcα-8NeuAcα-8NeuAcα-6Galβ-Cer36	28	130
20	Glucocerebrosides	Glcβ-Cer?	-	100
21	GSF-19	SU-6Glcβ-C30	-	-
22	GSC-60	NeuAcα-6Glcβ-Cer36	-	7
23	GSC-9	NeuAcα-(S)-6Glcβ-Cer36	-	-
24	GSC-62	NeuAcα-2Glcβ-Cer36	-	-
25	GSC-59	NeuAcα-6GlcNAcβ-Cer36	-	-
26	GSC-95	NeuAcα-(S)-6GlcNAcβ-Cer36	-	79
27	GSC-232	NeuAcα-8NeuAcα-6Glcβ-Cer36	66	-
28	Lactocerebrosides	Galβ-4Glcβ-Cer	-	108
29	Lac	Galβ-4Glc-DH	71	16
30	Lac-AO	Galβ-4Glc-AO	-	-
31	GSC-432	(3-deoxy,3-carboxymethyl)Galβ-4Glcβ-C30	-	-
32	GSC-296	GlcAβ-3Galβ-4Glcβ-C30	102	-
33	GSC-353	SU-3GlcAβ-3Galβ-4Glcβ-C30	-	-
34	GalNAcα-3Galß-4Glc	GalNAcα-3Galβ-4Glc-DH	192	170
35	Globotri-AO	Galα-4Galβ-4Glcβ-AO	-	-
36	Ceramide trihexoside	Galα-4Galβ-4Glcβ-Cer	-	49
37	Globoside (P-antigen)	GalNAcβ-3Galα-4Galβ-4Glcβ-Cer	-	-
38	Forssmann glycolipid	GalNAcα-3GalNAcβ-3Galα-4Galβ-4Glcβ-Cer	-	-
39	Fuc(3)-Lac-AO	Galβ-4Glc-AO<br>| Fucα-3	434	-
40	GSC-430	(3-deoxy,3-carboxymethyl)Galβ-3Glcβ-C30<br>| Fucα-4	204	-
41	GSC-260	(3-deoxy,3-carboxymethyl)Galβ-4Glcβ-C30<br>| Fucα-3	100	-
42	GSC-150	SU-3Galβ-4Glcβ-C30<br>| Fucα-3	-	-
43	GSC-160	SU-3Galβ-4Glcβ-Cer36<br>| Fucα-3	321	-
44	NeuAcα-(3')Lac	NeuAcα-3Galβ-4Glc-DH	-	50,438
45	NeuAcα-(3')Lac-AO	NeuAcα-3Galβ-4Glc-AO	-	16,272
46	Neu4,5Ac-(3')Lac	(4-OAc)NeuAcα-3Galβ-4Glc-DH	7,279	12,366
47	Neu4,5Ac-(3')Lac-AO	(4-OAc)NeuAcα-3Galβ-4Glc-AO	107	297
48	GSC-16	NeuAcα-3Galβ-4Glcβ-Cer32	110	72
49	GSC-178	NeuAcα-3Galβ-4Glcβ-Cer34	267	434
50	GSC-17	NeuAcα-3Galβ-4Glcβ-Cer36	207	189
51	GSC-18	NeuAcα-3Galβ-4Glcβ-Cer42	-	199

52	GSC-197	KDNα-3Galβ-4Glcβ-Cer28	-	-
53	GSC-199	KDNα-3Galβ-4Glcβ-C30	-	-
54	GSC-198	KDNα-3Galβ-4Glcβ-Cer34	-	-
55	GSC-75	(4-deoxy)NeuAcα-3Galβ-4Glcβ-Cer36	-	-
56	GSC-76	(7-deoxy)NeuAcα-3Galβ-4Glcβ-Cer36	27	-
57	GSC-77	(8-deoxy)NeuAcα-3Galβ-4Glcβ-Cer36	-	386
58	GSC-153	(4,8-deoxy)NeuAcα-3Galβ-4Glcβ-Cer36	-	17
59	GSC-51	(9-deoxy)NeuAcα-3Galβ-4Glcβ-Cer36	-	1,254
60	GSC-78	(4-OMe)NeuAcα-3Galβ-4Glcβ-Cer36	-	-
61	GSC-79	(9-OMe)NeuAcα-3Galβ-4Glcβ-Cer36	-	-
62	GSC-23	(C7)NeuAcα-3Galβ-4Glcβ-Cer36	167	-
63	GSC-24	(C8)NeuAcα-3Galβ-4Glcβ-Cer36	-	-
64	GSC-50	(C8 diastereoisomer)NeuAcα-3Galβ-4Glcβ-Cer36	-	1,414
65	GSC-229	NeuAcα-8NeuAcα-3Galβ-4Glcβ-Cer36	-	-
66	GSC-96	NeuAcα-9NeuAcα-3Galβ-4Glcβ-Cer36	478	2,230
67	GSC-437	NeuAcα-8NeuAcα-8NeuAcα-3Galβ-4Glcβ-Cer36	-	-
68	Neuα-(3')Lac	Neuα-3Galβ-4Glc-DH	696	238
69	Neuα-(3')Lac-AO	Neuα-3Galβ-4Glc-AO	224	-
70	NeuAcα-(6')Lac	NeuAcα-6Galβ-4Glc-DH	-	-
71	NeuAcα-(6')Lac-AO	NeuAcα-6Galβ-4Glc-AO	-	-
72	GSC-61	NeuAcα-6Galβ-4Glcβ-Cer36	-	-
73	GSC-12	NeuAcα-(S)-6Galβ-4Glcβ-Cer36	-	-
74	GSC-234	NeuAcα-(S)-6Gal(S)β-4Glcβ-Cer36	-	14
75	GSC-73	NeuAcα-(S)-6Galβ-4Glcβ-(S)-Cer36	-	-
76	Neuα-(6')Lac	Neuα-6Galβ-4Glc-DH	987	352
77	Neuα-(6')Lac-AO	Neuα-6Galβ-4Glc-AO	577	-
78	NeuAcß-(3')Lac	NeuAcβ-3Galβ-4Glc-DH	-	-
79	NeuAcß-(3')Lac-AO	NeuAcβ-3Galβ-4Glc-AO	-	-
80	NeuAcß-(6')Lac	NeuAcβ-6Galβ-4Glc-DH	161	64
81	NeuAcß-(6')Lac-AO	NeuAcβ-6Galβ-4Glc-AO	101	-
82	GSC-161	NeuAcα-3Galβ-4Glcβ-C30<br>| Fucα-3	-	-
83	GSC-162	NeuAcα-3Galβ-4Glcβ-Cer36<br>| Fucα-3	-	-
84	LacNAc(1-3)	Galβ-3GlcNAc-DH	68	-
85	LacNAc(1-3)-AO	Galβ-3GlcNAc-AO	334	-
86	LacNAc	Galβ-4GlcNAc-DH	-	-
87	LacNAc-AO	Galβ-4GlcNAc-AO	798	-
88	Galα-4Galß-4GlcNAc	Galα-4Galβ-4GlcNAc-DH	-	-
89	SU(3')-LN	SU-3Galβ-4GlcNAc-DH	-	-
90	Lea-Tri	Galβ-3GlcNAc-DH<br>| Fucα-4	-	3
91	Lea-Tri-AO	Galβ-3GlcNAc-AO<br>| Fucα-4	78	-
92	Lex-Tri	Galβ-4GlcNAc-DH<br>| Fucα-3	170	-
93	Lex-Tri-AO	Galβ-4GlcNAc-AO<br>| Fucα-3	622	-
94	Lex-Tri-(Me)AO	Galβ-4GlcNAc-(Me)AO<br>| Fucα-3	-	-
95	SU(3')-Lea-Tri	SU-3Galβ-3GlcNAc-DH<br>| Fucα-4	-	-
96	SU(3')-Lex-Tri	SU-3Galβ-4GlcNAc-DH<br>| Fucα-3	-	-
97	NeuAcα-(3')LN1-3	NeuAcα-3Galβ-3GlcNAc-DH	-	-
98	NeuAcα-(3')LN1-3-AO	NeuAcα-3Galβ-3GlcNAc-AO	-	-
99	NeuAcα-(3')LN	NeuAcα-3Galβ-4GlcNAc-DH	-	28,401
100	NeuAcα-(3')LN-AO	NeuAcα-3Galβ-4GlcNAc-AO	-	17,077
101	PI-1	NeuAcα-3(6-NAc)Galβ-4GlcNAc-DH	-	2,211
102	PI-1-AO	NeuAcα-3(6-NAc)Galβ-4GlcNAc-AO	-	24,239
103	PI-2	NeuAcα-3(6-NBz)Galβ-4GlcNAc-DH	-	31,471
104	PI-2-AO	NeuAcα-3(6-NBz)Galβ-4GlcNAc-AO	-	22,452
105	NeuAcα-(6')LN	NeuAcα-6Galβ-4GlcNAc-DH	-	-

Supporting information

106	NeuAcα-(6')LN-AO	NeuAcα-6Galβ-4GlcNAc-AO	-	-
107	Neu5,9Ac-(6')LN	(9-OAc)NeuAcα-6Galβ-4GlcNAc-DH	-	-
108	SA(3')-Lea-Tri	NeuAcα-3Galβ-3GlcNAc-DH<br>| Fucα-4	-	-
109	SA(3')-Lea-Tri-AO	NeuAcα-3Galβ-3GlcNAc-AO<br>| Fucα-4	-	-
110	SA(3')-Lex-Tri	NeuAcα-3Galβ-4GlcNAc-DH<br>| Fucα-3	-	1,847
111	SA(3')-Lex-Tri-AO	NeuAcα-3Galβ-4GlcNAc-AO<br>| Fucα-3	28	-
112	GSC-440	NeuAcα-3Galβ-4GlcNAcβ-C30<br>| Fucα-3	-	-
113	GSC-512	(4-OAc)NeuAcα-3Galβ-4GlcNAcβ-C30<br>| Fucα-3	-	-
114	GSC-513	(9-OAc)NeuAcα-3Galβ-3GlcNAcβ-C30<br>| Fucα-4	-	-
115	GSC-511	(9-OAc)NeuAcα-3Galβ-4GlcNAcβ-C30<br>| Fucα-3	-	-
116	GSC-225	(3-deoxy,3-carboxymethyl)Galβ-4GlcNAcβ-3Galβ-Cer36<br>| Fucα-3	-	-
117	GSC-236	SU-3Galβ-4GlcNAcβ-3Galβ-C30<br>| Fucα-3	-	-
118	GSC-479	NeuAcα-3Galβ-4GlcNAcβ-3Galβ-C30<br>| Fucα-3	-	-
119	GSC-105	NeuAcα-3Galβ-4GlcNAcβ-3Galβ-Cer36<br>| Fucα-3	92	-
120	GSC-121	NeuAcα-3Galβ-4GlcNAcβ-3Galβ-Cer36<br>| (3-deoxy)Fucα-3	458	-
121	GSC-123	NeuAcα-3Galβ-4GlcNAcβ-3Galβ-Cer36<br>| (4-deoxy)Fucα-3	-	-
122	GSC-133	NeuAcα-3Galβ-4GlcNAcβ-3Galβ-Cer36<br>| (2-OMe)Fucα-3	-	-
123	GSC-131	NeuAcα-3Galβ-4GlcNAcβ-3Galβ-Cer36<br>| Quvα-3	-	-
124	GSC-163	NeuAcα-3Galβ-4GlcNAcβ-3Galβ-Cer36<br>| Rhaα-3	-	-
125	GSC-127	NeuAcα-3Galβ-4GlcNAcβ-3Galβ-Cer36<br>| (6-deoxy)Talα-3	-	-
126	GSC-341	KDNα-3Galβ-4GlcNAcβ-3Galβ-C30<br>| Fucα-3	-	-
127	GSC-177	NeuGcα-3Galβ-4GlcNAcβ-3Galβ-Cer36<br>| Fucα-3	217	-
128	GSC-175	NeuAcα-3(4-deoxy)Galβ-4GlcNAcβ-3Galβ-Cer36<br>| Fucα-3	-	25
129	GSC-176	NeuAcα-3(6-deoxy)Galβ-4GlcNAcβ-3Galβ-Cer36<br>| Fucα-3	-	-
130	GSC-257	NeuAcα-3(4,6-deoxy)Galβ-4GlcNAcβ-3Galβ-Cer36<br>| Fucα-3	-	-
131	DLNN	GlcNAcβ-3Galβ-4Glc-DH	-	144
132	LNT	Galβ-3GlcNAcβ-3Galβ-4Glc-DH	-	-
133	Paragloboside	Galβ-4GlcNAcβ-3Galβ-4Glcβ-Cer	-	-
134	LNnT	Galβ-4GlcNAcβ-3Galβ-4Glc-DH	-	-
135	B-like pentaosylceramide	Galα-3Galβ-4GlcNAcβ-3Galβ-4Glcβ-Cer	-	-
136	Klaus glycolipid	Galβ-3Galβ-4GlcNAcβ-3Galβ-4Glcβ-Cer	-	-
137	GSC-207	GlcAβ-3Galβ-4GlcNAcβ-3Galβ-4Glcβ-C30	25	-
138	GSC-191	GlcAβ-3Galβ-4GlcNAcβ-3Galβ-4Glcβ-Cer36	-	-
139	GSC-189	GlcAβ-3Galβ-4GlcNAcβ-3Galβ-4Glcβ-Cer42	-	-
140	SU(3')-Tri	SU-3Galβ-4GlcNAcβ-3Gal-DH	-	-
141	GSC-208	SU-3GlcAβ-3Galβ-4GlcNAcβ-3Galβ-4Glcβ-C30	34	-
142	GSC-192	SU-3GlcAβ-3Galβ-4GlcNAcβ-3Galβ-4Glcβ-Cer36	-	-
143	GSC-190	SU-3GlcAβ-3Galβ-4GlcNAcβ-3Galβ-4Glcβ-Cer42	118	-
144	Led-II pentaosylceramide	Fucα-2Galβ-3GlcNAcβ-3Galβ-4Glcβ-CerA	-	-
145	Led-I pentaosylceramide	Fucα-2Galβ-3GlcNAcβ-3Galβ-4Glcβ-CerB	-	59
146	LNFP-I	Fucα-2Galβ-3GlcNAcβ-3Galβ-4Glc-DH	-	-

Supporting information

147	B-hexaosylceramide	Galα-3Galβ-4GlcNAcβ-3Galβ-4Glcβ-Cer<br>Fucα-2	-	-
148	A-Hexa	GalNAcα-3Galβ-3GlcNAcβ-3Galβ-4Glc-DH<br>Fucα-2	25	-
149	A-Hepta	Fucα-4<br>GalNAcα-3Galβ-3GlcNAcβ-3Galβ-4Glc-DH<br>Fucα-2	-	-
150	LNFP-II	Galβ-3GlcNAcβ-3Galβ-4Glc-DH<br>Fucα-4	83	-
151	LNDFH-II	Galβ-3GlcNAcβ-3Galβ-4Glc-DH<br>Fucα-4 Fucα-3	-	-
152	Leb-hexaosylceramide	Fucα-2Galβ-3GlcNAcβ-3Galβ-4Glcβ-Cer<br>Fucα-4	-	-
153	LNDFH-I	Fucα-2Galβ-3GlcNAcβ-3Galβ-4Glc-DH<br>Fucα-4	168	-
154	LNTFH-I	Fucα-2Galβ-3GlcNAcβ-3Galβ-4Glc-DH<br>Fucα-4 Fucα-2	-	-
155	LNFP-III	Galβ-4GlcNAcβ-3Galβ-4Glc-DH<br>Fucα-3	-	-
156	LNFP-III-AO	Galβ-4GlcNAcβ-3Galβ-4Glc-AO<br>Fucα-3	-	-
157	LNnDFH-I	Fucα-2Galβ-4GlcNAcβ-3Galβ-4Glc-DH<br>Fucα-3	32	-
158	LNnDFH-II	Galβ-4GlcNAcβ-3Galβ-4Glc-DH<br>Fucα-3 Fucα-3	-	-
159	LNnDFH-V	Galβ-4GlcNAcβ-3Galβ-4Glc-DH<br>Fucα-3 Fucα-2	-	711
160	LNnTFH-I	Fucα-2Galβ-4GlcNAcβ-3Galβ-4Glc-DH<br>Fucα-3 Fucα-2	-	24
161	SU(3')-LNFP-II	SU-3Galβ-3GlcNAcβ-4Galβ-4Glc-DH<br>Fucα-4	-	-
162	SU(6')-LNFP-II	SU-6Galβ-3GlcNAcβ-3Galβ-4Glc-DH<br>Fucα-4	-	-
163	SU(3')-LNFP-III	SU-3Galβ-4GlcNAcβ-3Galβ-4Glc-DH<br>Fucα-3	-	-
164	SU(6')-LNFP-III	SU-6Galβ-4GlcNAcβ-3Galβ-4Glc-DH<br>Fucα-3	-	-
165	SU(3',6)-LNFP-III	SU-6<br>SU-3Galβ-4GlcNAcβ-3Galβ-4Glc-DH<br>Fucα-3	-	140
166	LSTa	NeuAcα-3Galβ-3GlcNAcβ-3Galβ-4Glc-DH	-	48
167	GSC-272	NeuAcα-3Galβ-3GlcNAcβ-3Galβ-4Glcβ-C30	-	-
168	GSC-147	KDNα-3Galβ-3GlcNAcβ-3Galβ-4Glcβ-Cer36	-	-
169	GSC-396	NeuGcα-3Galβ-3GlcNAcβ-3Galβ-4Glcβ-C30	-	-
170	LSTb	Galβ-3GlcNAcβ-3Galβ-4Glc-DH<br>NeuAcα-6	-	-
171	GSC-397	NeuGcα-6Galβ-3GlcNAcβ-3Galβ-4Glcβ-C30	-	-
172	DSLNT	NeuAcα-3Galβ-3GlcNAcβ-3Galβ-4Glc-DH<br>NeuAcα-6	-	302
173	Sialylparagloboside	NeuAcα-3Galβ-4GlcNAcβ-3Galβ-4Glcβ-Cer	34	-
174	GSC-273	NeuAcα-3Galβ-4GlcNAcβ-3Galβ-4Glcβ-C30	-	23,862
175	GSC-31	NeuAcα-3Galβ-4GlcNAcβ-3Galβ-4Glcβ-Cer36	-	-
176	LSTc	NeuAcα-6Galβ-4GlcNAcβ-3Galβ-4Glc-DH	-	-
177	GSC-516B	Neuα-3Galβ-4GlcNAcβ-3Galβ-4Glcβ-Cer36<br>SU-6	61	-
178	SA(3/6)LNFP-I	NeuAcα-3/6Galβ-3GlcNAcβ-3Galβ-4Glc-DH<br>Fucα-2	-	-
179	SA(3')-LNFP-II	NeuAcα-3Galβ-3GlcNAcβ-3Galβ-4Glc-DH<br>Fucα-4	-	-
180	SA(6')-LNFP-VI	NeuAcα-6Galβ-4GlcNAcβ-3Galβ-4Glc-DH<br>Fucα-3	-	-
181	GSC-533	NeuAcα-3Galβ-4GlcNβ-3Galβ-4Glcβ-Cer36<br>Fucα-3	-	100
182	GSC-64	NeuAcα-3Galβ-4GlcNAcβ-3Galβ-4Glcβ-Cer36<br>Fucα-3	-	-
183	SA(3')-LNFP-III	NeuAcα-3Galβ-4GlcNAcβ-3Galβ-4Glc-DH<br>Fucα-3	-	-

Supporting information

184	GSC-472	Neuα-3Galβ-4GlcNAcβ-3Galβ-4Glcβ-Cer36<br>Fucα-3	137	-
185	GSC-97	NeuAcα-6Galβ-4GlcNAcβ-3Galβ-4Glcβ-Cer36<br>Fucα-3	-	422
186	GSC-314	KDNα-3Galβ-4GlcNAcβ-3Galβ-4Glcβ-C30<br>Fucα-3	8	33
187	GSC-149	KDNα-3Galβ-4GlcNAcβ-3Galβ-4Glcβ-Cer36<br>Fucα-3	-	-
188	GSC-311	KDNα-3Galβ-4GlcNAcβ-3Galβ-4Glcβ-C30<br>Rhaα-3	-	-
189	GSC-268	SU-6<br>NeuAcα-3Galβ-4GlcNAcβ-3Galβ-4Glcβ-Cer36<br>Fucα-3	-	6,909
190	GSC-268 deNAc	SU-6<br>Neuα-3Galβ-4GlcNβ-3Galβ-4Glcβ-Cer36<br>Fucα-3	194	209
191	GSC-269	SU-6<br>NeuAcα-3Galβ-4GlcNAcβ-3Galβ-4Glcβ-Cer36<br>Fucα-3	-	-
192	GSC-406	SU-6<br>Neuα-3Galβ-4GlcNAcβ-3Galβ-4Glcβ-Cer36<br>Fucα-3	91	-
193	GSC-270	SU-6 SU-6<br>NeuAcα-3Galβ-4GlcNAcβ-3Galβ-4Glcβ-Cer36<br>Fucα-3	-	17,163
194	pLNH	Galβ-3GlcNAcβ-3Galβ-4GlcNAcβ-3Galβ-4Glc-DH	79	26
195	pLNnH	Galβ-4GlcNAcβ-3Galβ-4GlcNAcβ-3Galβ-4Glc-DH	-	-
196	GSC-216	GlcAβ-3Galβ-4GlcNAcβ-3Galβ-4GlcNAcβ-3Galβ-4Glcβ-Cer42	-	-
197	GSC-217	SU-3GlcAβ-3Galβ-4GlcNAcβ-3Galβ-4GlcNAcβ-3Galβ-4Glcβ-Cer42	-	-
198	GSC-218	GlcAβ-3Galβ-4GlcNAcβ-3Galβ-4GlcNAcβ-3Galβ-4Glcβ-Cer36	215	89
199	GSC-219	SU-3GlcAβ-3Galβ-4GlcNAcβ-3Galβ-4GlcNAcβ-3Galβ-4Glcβ-Cer36	-	-
200	LNH	Galβ-4GlcNAcβ-6<br>Galβ-4Glc-DH<br>Galβ-3GlcNAcβ-3	-	-
201	iLNO	Galβ-3GlcNAcβ-3Galβ-4GlcNAcβ-6<br>Galβ-4Glc-DH<br>Galβ-3GlcNAcβ-3	-	-
202	LND	Galβ-4GlcNAcβ-6<br>Galβ-4GlcNAcβ-6<br>Galβ-3GlcNAcβ-3 Galβ-4Glc-DH<br>Galβ-3GlcNAcβ-3	-	-
203	LNnH	Galβ-4GlcNAcβ-6<br>Galβ-4Glc-DH<br>Galβ-4GlcNAcβ-3	-	-
204	Nonaosylceramide	GlcNAcβ-6<br>GlcNAcβ-6 Galβ-4GlcNAcβ-3Galβ-4Glcβ-Cer<br>Galβ-4GlcNAcβ-3<br>GlcNAcβ-3	-	-
205	I-octaosylceramide	Galβ-4GlcNAcβ-6<br>Galβ-4GlcNAcβ-3Galβ-4Glcβ-Cer<br>Galβ-4GlcNAcβ-3	-	-
206	I-dodecaosylceramide	Galβ-4GlcNAcβ-6<br>Galβ-4GlcNAcβ-6 Galβ-4GlcNAcβ-3Galβ-4Glcβ-Cer<br>Galβ-4GlcNAcβ-3<br>Galβ-4GlcNAcβ-3	-	-
207	I-hexadecaosylceramide	Galβ-4GlcNAcβ-6<br>Galβ-4GlcNAcβ-6 Galβ-4GlcNAcβ-3Galβ-4Glcβ-Cer<br>Galβ-4GlcNAcβ-6 Galβ-4GlcNAcβ-3<br>Galβ-4GlcNAcβ-3<br>Galβ-4GlcNAcβ-3	-	-

Supporting information

208	I-eicosaosylceramide	Galβ-4GlcNAcβ-6<br>Galβ-4GlcNAcβ-6 Galβ-4GlcNAcβ-3Galβ-4Glcβ-Cer<br>Galβ-4GlcNAcβ-6 Galβ-4GlcNAcβ-3<br>Galβ-4GlcNAcβ-6 Galβ-4GlcNAcβ-3<br>Galβ-4GlcNAcβ-3<br>Galβ-4GlcNAcβ-3	-	-
209	B-like decaosylceramide	Galα-3Galβ-4GlcNAcβ-6<br>Galβ-4GlcNAcβ-3Galβ-4Glcβ-Cer<br>Galα-3Galβ-4GlcNAcβ-3	-	-
210	B-like pentadecaosylcera	Galα-3Galβ-4GlcNAcβ-6<br>Galα-3Galβ-4GlcNAcβ-6 Galβ-4GlcNAcβ-3Galβ-4Glcβ-Cer<br>Galβ-4GlcNAcβ-3<br>Galα-3Galβ-4GlcNAcβ-3	-	76
211	B-like eicosaosylceramide	Galα-3Galβ-4GlcNAcβ-6<br>Galα-3Galβ-4GlcNAcβ-6 Galβ-4GlcNAcβ-3Galβ-4Glcβ-Cer<br>Galα-3Galβ-4GlcNAcβ-6 Galβ-4GlcNAcβ-3<br>Galβ-4GlcNAcβ-3<br>Galα-3Galβ-4GlcNAcβ-3	-	-
212	B-like pentaeicosaosylcer	Galα-3Galβ-4GlcNAcβ-6<br>Galα-3Galβ-4GlcNAcβ-6 Galβ-4GlcNAcβ-3Galβ-4Glcβ-Cer<br>Galα-3Galβ-4GlcNAcβ-6 Galβ-4GlcNAcβ-3<br>Galα-3Galβ-4GlcNAcβ-6 Galβ-4GlcNAcβ-3<br>Galβ-4GlcNAcβ-3<br>Galα-3Galβ-4GlcNAcβ-3	-	4
213	pLNFH-IV	Galβ-3GlcNAcβ-3Galβ-4GlcNAcβ-3Galβ-4Glc-DH<br>Fucα-3	-	-
214	DFpLNH-II	Galβ-3GlcNAcβ-3Galβ-4GlcNAcβ-3Galβ-4Glc-DH<br>Fucα-4 Fucα-3	-	-
215	TFpLNH-I	Fucα-2Galβ-3GlcNAcβ-3Galβ-4GlcNAcβ-3Galβ-4Glc-DH<br>Fucα-4 Fucα-3	-	-
216	MFLNH-III	Galβ-4GlcNAcβ-6<br>Fucα-3 Galβ-4Glc-DH<br>Galβ-3GlcNAcβ-3	-	-
217	DFLNH(b)	Galβ-4GlcNAcβ-6<br>Fucα-3 Galβ-4Glc-DH<br>Galβ-3GlcNAcβ-3<br>Fucα-4	-	-
218	DFLNH(c)	Galβ-4GlcNAcβ-6<br>Galβ-4Glc-DH<br>Fucα-2Galβ-3GlcNAcβ-3<br>Fucα-4	-	-
219	DFLNH(a)	Galβ-4GlcNAcβ-6<br>Fucα-3 Galβ-4Glc-DH<br>Fucα-2Galβ-3GlcNAcβ-3	-	-
220	TFLNH	Galβ-4GlcNAcβ-6<br>Fucα-3 Galβ-4Glc-DH<br>Fucα-2Galβ-3GlcNAcβ-3<br>Fucα-4	21	-
221	MFiLNO-IV	Galβ-3GlcNAcβ-3Galβ-4GlcNAcβ-6<br>Fucα-3 Galβ-4Glc-DH<br>Galβ-3GlcNAcβ-3	-	-
222	TFiLNO	Galβ-3GlcNAcβ-3Galβ-4GlcNAcβ-6<br>Fucα-4 Fucα-3 Galβ-4Glc-DH<br>Galβ-3GlcNAcβ-3<br>Fucα-4	79	-
223	MFLND	Galβ-4GlcNAcβ-6<br>Fucα-3 Galβ-4GlcNAcβ-6<br>Galβ-3GlcNAcβ-3 Galβ-4Glc-DH<br>Galβ-3GlcNAcβ-3	-	-

Supporting information

224	MFLNnH(a)	Galβ-4GlcNAcβ-6<br>Fucα-3 Galβ-4Glc-DH<br>Galβ-4GlcNAcβ-3	-	-
225	DFLNnH	Galβ-4GlcNAcβ-6<br>Fucα-3 Galβ-4Glc-DH<br>Galβ-4GlcNAcβ-3<br>Fucα-3	-	-
226	B-III dodecaosylceramide	Galα-3Galβ-4GlcNAcβ-6<br>Fucα-2 Galβ-4GlcNAcβ-3Galβ-4Glcβ-Cer<br>Galα-3Galβ-4GlcNAcβ-3<br>Fucα-2	-	-
227	B-IV tetradecaosylcerami	Galα-3Galβ-4GlcNAcβ-6<br>Fucα-2 Galβ-4GlcNAcβ-3Galβ-4Glcβ-Cer<br>Galα-3Galβ-4GlcNAcβ-3Galβ-4GlcNAcβ-3<br>Fucα-2	-	-
228	MSLNH	NeuAcα-6Galβ-4GlcNAcβ-6<br>Galβ-4Glc-DH<br>Galβ-3GlcNAcβ-3	-	-
229	MSLNnH-I	Galβ-4GlcNAcβ-6<br>Galβ-4Glc-DH<br>NeuAcα-6Galβ-3GlcNAcβ-3	-	54
230	DSLNnH	NeuAcα-6Galβ-4GlcNAcβ-6<br>Galβ-4Glc-DH<br>NeuAcα-6Galβ-4GlcNAcβ-3	-	8
231	MSMFLNH	Galβ-4GlcNAcβ-6<br>Fucα-3 Galβ-4Glc-DH<br>NeuAcα-3Galβ-3GlcNAcβ-3	-	-
232	MFMSLNnH	Galβ-4GlcNAcβ-6<br>Fucα-3 Galβ-4Glc-DH<br>NeuAcα-6Galβ-3GlcNAcβ-3	-	-
233	GSC-221	NeuAcα-3Galβ-4GlcNAcβ-3Galβ-4GlcNAcβ-3Galβ-4Glcβ-Cer36<br>Fucα-3	-	-
234	GSC-220	NeuAcα-3Galβ-4GlcNAcβ-3Galβ-4GlcNAcβ-3Galβ-4Glcβ-Cer36<br>Fucα-3 Fucα-3	-	-
235	C4U	NeuAcα-3Galβ-4GlcNAcβ-3Galβ-3GlcNAc-DH<br>SU-6 SU-6 SU-6	-	49,773
236	Man2(α2)	Manα-2Man-DH	11	87
237	Man2(α3)	Manα-3Man-DH	151	128
238	Man2(α6)	Manα-6Man-DH	133	143
239	Man3(α3,α6)	Manα-6Man-DH<br>Manα-3	88	51
240	Man5(α3,α6)	Manα-3<br>Manα-6Manα-6Man-DH<br>Manα-3	116	6
241	Man1GN1	Manβ-4GlcNAc-DH	10	-
242	Man2GN1	Manα-3Manβ-4GlcNAc-DH	31	81
243	Man2aGN2	Manα-6Manβ-4GlcNAcβ-4GlcNAc-DH	-	-
244	Man3GN2	Manα-6<br>Manβ-4GlcNAcβ-4GlcNAc-DH<br>Manα-3	-	12
245	Man4aGN2	Manα-3Manα-6<br>Manβ-4GlcNAcβ-4GlcNAc-DH<br>Manα-3	-	-
246	Man4bGN2	Manα-6<br>Manα-3Manα-6<br>Manβ-4GlcNAcβ-4GlcNAc-DH	-	-
247	Man5GN2	Manα-6<br>Manα-3Manα-6<br>Manβ-4GlcNAcβ-4GlcNAc-DH<br>Manα-3	-	-

248	Man6GN2	Manα-6<br>Manα-3Manα-6<br>Manβ-4GlcNAcβ-4GlcNAc-DH<br>Manα-2Manα-3	66	-
249	Man7(D1)GN2	Manα-6<br>Manα-3Manα-6<br>Manβ-4GlcNAcβ-4GlcNAc-DH<br>Manα-2Manα-2Manα-3	55	-
250	Man7(D1)GN2-AO	Manα-6<br>Manα-3Manα-6<br>Manβ-4GlcNAcβ-4GlcNAc-AO<br>Manα-2Manα-2Manα-3	-	-
251	Man7(D3)GN2	Manα-2Manα-6<br>Manα-3Manα-6<br>Manβ-4GlcNAcβ-4GlcNAc-DH<br>Manα-2Manα-3	-	-
252	Man8(D1D3)GN2	Manα-2Manα-6<br>Manα-3Manα-6<br>Manβ-4GlcNAcβ-4GlcNAc-DH<br>Manα-2Manα-2Manα-3	-	-
253	Man9GN2	Manα-2Manα-6<br>Manα-2Manα-3Manα-6<br>Manβ-4GlcNAcβ-4GlcNAc-DH<br>Manα-2Manα-2Manα-3	24	-
254	Man9GN2-AO	Manα-2Manα-6<br>Manα-2Manα-3Manα-6<br>Manβ-4GlcNAcβ-4GlcNAc-AO<br>Manα-2Manα-2Manα-3	-	-
255	Glc1Man9GN2	Manα-2Manα-6<br>Manα-6<br>Manα-2Manα-3 Manβ-4GlcNAcβ-4GlcNAc-DH<br>Glcα-3Manα-2Manα-2Manα-3	-	-
256	Glc1Man9GN2-AO	Manα-2Manα-6<br>Manα-6<br>Manα-2Manα-3 Manβ-4GlcNAcβ-4GlcNAc-AO<br>Glcα-3Manα-2Manα-2Manα-3	-	-
257	Glc2Man9GN2-AO	Manα-2Manα-6<br>Manα-6<br>Manα-2Manα-3 Manβ-4GlcNAcβ-4GlcNAc-AO<br>Glcα-3Glcα-3Manα-2Manα-2Manα-3	-	-
258	Glc2Man7(D1)GN1-AO	Manα-6<br>Manα-3Manα-6<br>Manβ-4GlcNAc-AO<br>Glcα-3Glcα-3Manα-2Manα-2Manα-3	-	-
259	Glc3Man7(D1)GN1-AO	Manα-6<br>Manα-3Manα-6<br>Manβ-4GlcNAc-AO<br>Glcα-2Glcα-3Glcα-3Manα-2Manα-2Manα-3	-	-
260	Man3XylGN2	Manα-6<br>Xylβ-2Manβ-4GlcNAcβ-4GlcNAc-DH<br>Manα-3	-	-
261	N1	Galβ-4GlcNAcβ-2Manα-6 Fucα-6<br>Manβ-4GlcNAcβ-4GlcNAc-DH<br>Manα-3	-	-
262	N2	Manα-6<br>Manβ-4GlcNAcβ-4GlcNAc-DH<br>Galβ-4GlcNAcβ-2Manα-3	-	-
263	N4	Galβ-4GlcNAcβ-2Manα-6<br>Manβ-4GlcNAcβ-4GlcNAc-DH<br>Manα-3	-	-

Supporting information

264	GlcNac2Man3-AO	GlcNAcβ-2Manα-6<br>Man-AO<br>GlcNAcβ-2Manα-3	-	-
265	N3	GlcNAcβ-2Manα-6 Fucα-6<br>Galβ-4 Manβ-4GlcNAcβ-4GlcNAc-DH<br>GlcNAcβ-2Manα-3	-	-
266	NGA2	GlcNAcβ-2Manα-6<br>Manβ-4GlcNAcβ-4GlcNAc-DH<br>GlcNAcβ-2Manα-3	16	-
267	NGA2B	GlcNAcβ-2Manα-6<br>GlcNAcβ-4Manβ-4GlcNAcβ-4GlcNAc-DH<br>GlcNAcβ-2Manα-3	-	36
268	NGA3B	GlcNAcβ-2Manα-6<br>GlcNAcβ-4Manβ-4GlcNAcβ-4GlcNAc-DH<br>GlcNAcβ-4Manα-3<br>GlcNAcβ-2	-	-
269	NGA4	GlcNAcβ-6<br>GlcNAcβ-2Manα-6<br>Manβ-4GlcNAcβ-4GlcNAc-DH<br>GlcNAcβ-2Manα-3<br>GlcNAcβ-4	-	-
270	NGA5B	GlcNAcβ-2<br>GlcNAcβ-4Manα-6<br>GlcNAcβ-6<br>GlcNAcβ-4Manβ-4GlcNAcβ-4GlcNAc-DH<br>GlcNAcβ-4Manα-3<br>GlcNAcβ-2	2	-
271	GNMan5BGN2	Manα-6<br>Manα-3Manα-6<br>GlcNAcβ-4Manβ-4GlcNAcβ-4GlcNAc-DH<br>GlcNAcβ-2Manα-3	-	-
272	NA2	Galβ-4GlcNAcβ-2Manα-6<br>Manβ-4GlcNAcβ-4GlcNAc-DH<br>Galβ-4GlcNAcβ-2Manα-3	-	-
273	NA3	Galβ-4GlcNAcβ-2Manα-6<br>Manβ-4GlcNAcβ-4GlcNAc-DH<br>Galβ-4GlcNAcβ-4Manα-3<br>Galβ-4GlcNAcβ-2	-	-
274	NA4	Galβ-4GlcNAcβ-6<br>Galβ-4GlcNAcβ-2Manα-6<br>Manβ-4GlcNAcβ-4GlcNAc-DH<br>Galβ-4GlcNAcβ-4Manα-3<br>Galβ-4GlcNAcβ-2	18	-
275	Fuc-GlcNAc	Fucα-6GlcNAc-DH	37	-
276	Man3FGN2	Manα-6 Fucα-6<br>Manβ-4GlcNAcβ-4GlcNAc-DH<br>Manα-3	-	-
277	Man3FXylGN2	Manα-6<br>Xylβ-2Manα-4GlcNAcβ-4GlcNAc-DH<br>Manα-3 Fucα-3	-	-
278	NGA2F	GlcNAcβ-2Manα-6 Fucα-6<br>Manβ-4GlcNAcβ-4GlcNAc-DH<br>GlcNAcβ-2Manα-3	86	-
279	NA2F	Galβ-4GlcNAcβ-2Manα-6 Fucα-6<br>Manβ-4GlcNAcβ-4GlcNAc-DH<br>Galβ-4GlcNAcβ-2Manα-3	-	-
280	NA2F-AO	Galβ-4GlcNAcβ-2Manα-6 Fucα-6<br>Manβ-4GlcNAcβ-4GlcNAc-AO<br>Galβ-4GlcNAcβ-2Manα-3	-	-
281	NA2FB	Galβ-4GlcNAcβ-2Manα-6 Fucα-6<br>GlcNAcβ-4Manβ-4GlcNAcβ-4GlcNAc-DH<br>Galβ-4GlcNAcβ-2Manα-3	-	-

282	NA3-Lex	Galβ-4GlcNAcβ-2Manα-6<br>Manβ-4GlcNAcβ-4GlcNAc-DH<br>Galβ-4GlcNAcβ-4Manα-3<br>Galβ-4GlcNAcβ-2	-	-
283	A2(2-6)	NeuAcα-6Galβ-4GlcNAcβ-2Manα-6<br>Manβ-4GlcNAcβ-4GlcNAc-DH<br>NeuAcα-6Galβ-4GlcNAcβ-2Manα-3	-	-
284	AGP-Bi-Ac2	NeuAcα-6Galβ-4GlcNAcβ-2Manα-6<br>Manβ-4GlcNAcβ-4GlcNAc-DH<br>NeuAcα-6Galβ-4GlcNAcβ-2Manα-3	-	-
285	AGP-Bi-Gc2	NeuGcα-6Galβ-4GlcNAcβ-2Manα-6<br>Manβ-4GlcNAcβ-4GlcNAc-DH<br>NeuGcα-6Galβ-4GlcNAcβ-2Manα-3	-	-
286	AGP-Bi-AcGc	NeuGcα-6Galβ-4GlcNAcβ-2Manα-6<br>Manβ-4GlcNAcβ-4GlcNAc-DH<br>NeuAcα-6Galβ-4GlcNAcβ-2Manα-3	-	-
287	A3	NeuAcα-3Galβ-4GlcNAcβ-2Manα-6<br>Manβ-4GlcNAcβ-4GlcNAc-DH<br>NeuAcα-3Galβ-4GlcNAcβ-4Manα-3<br>NeuAcα-6Galβ-4GlcNAcβ-2	-	-
288	A2F(2-3)	NeuAcα-3Galβ-4GlcNAcβ-2Manα-6 Fucα-6<br>Manβ-4GlcNAcβ-4GlcNAc-DH<br>NeuAcα-3Galβ-4GlcNAcβ-2Manα-3	-	7,190
289	GM4	NeuAcα-3Galβ-Cer	-	-
290	SM3	SU-3Galβ-4Glcβ-Cer	-	-
291	Haematoside	NeuAcα-3Galβ-4Glcβ-Cer	-	1,399
292	GM3	NeuAcα-3Galβ-4Glcβ-Cer	-	223
293	GM3(Gc)	NeuGcα-3Galβ-4Glc-Cer	-	-
294	Asialo-GM2	GalNAcβ-4Galβ-4Glcβ-Cer	-	68
295	SM2	GalNAcβ-4Galβ-4Glcβ-Cer<br>SU-3	-	106
296	SB2	SU-3GalNAcβ-4Galβ-4Glcβ-Cer<br>SU-3	-	-
297	GM2	GalNAcβ-4Galβ-4Glcβ-Cer<br>NeuAcα-3	-	-
298	GSC-576	GalNAcβ-4Galβ-3Glcβ-C30<br>NeuAcα-3	-	-
299	GSC-108	GalNAcβ-4Galβ-4Glcβ-Cer36<br>NeuAcα-3	-	-
300	GSC-193	GalNAcβ-4Galβ-4Glcβ-Cer36<br>KDNα-3	-	-
301	Asialo-GM1	Galβ-3GalNAcβ-4Galβ-4Glcβ-Cer	-	179
302	Asialo-GM1-Tetra	Galβ-3GalNAcβ-4Galβ-4Glc-DH	-	79
303	SM1a	Galβ-3GalNAcβ-4Galβ-4Glcβ-Cer<br>SU-3	-	-
304	SB1a	SU-3Galβ-3GalNAcβ-4Galβ-4Glcβ-Cer<br>SU-3	-	-
305	GSC-335	SU-6<br>NeuAcα-3Galβ-3GalNAcβ-4Galβ-4Glcβ-Cer36	-	-
306	GM1	Galβ-3GalNAcβ-4Galβ-4Glcβ-Cer<br>NeuAcα-3	-	-
307	GM1-penta	Galβ-3GalNAcβ-4Galβ-4Glc-DH<br>NeuAcα-3	-	-
308	GM1(Gc)	Galβ-3GalNAcβ-4Galβ-4Glcβ-Cer<br>NeuGcα-3	-	-
309	GM1(Gc)-penta	Galβ-3GalNAcβ-4Galβ-4Glc-DH<br>NeuGcα-3	5	-
310	GD1a	NeuAcα-3Galβ-3GalNAcβ-4Galβ-4Glcβ-Cer<br>NeuAcα-3	-	-
311	GD1a-hexa	NeuAcα-3Galβ-3GalNAcβ-4Galβ-4Glc-DH<br>NeuAcα-3	-	15,425
312	GalNAc-GD1a(Ac,Gc)	GalNAcβ-4Galβ-3GalNAcβ-4Galβ-4Glcβ-Cer<br>NeuGcα-3 NeuAcα-3<br>GalNAcβ-4Galβ-3GalNAcβ-4Galβ-4Glcβ-Cer<br>NeuAcα-3 NeuGcα-3	-	-

313	GSC-195	KDNα-3Galβ-3GalNAcβ-4Galβ-4Glcβ-Cer36<br>KDNα-3	24	102
314	GD3	NeuAcα-8NeuAcα-3Galβ-4Glcβ-Cer	-	-
315	GD3-tetra	NeuAcα-8NeuAcα-3Galβ-4Glc-DH	-	-
316	GD3-tetra-AO	NeuAcα-8NeuAcα-3Galβ-4Glc-AO	-	-
317	GD2	GalNAcβ-4Galβ-4Glcβ-Cer<br>NeuAcα-8NeuAcα-3	-	-
318	GD1b	Galβ-3GalNAcβ-4Galβ-4Glcβ-Cer<br>NeuAcα-8NeuAcα-3	-	-
319	GT1a	NeuAcα-8NeuAcα-3Galβ-3GalNAcβ-4Galβ-4Glcβ-Cer<br>NeuAcα-3	-	-
320	GT1b	NeuAcα-3Galβ-3GalNAcβ-4Galβ-4Glcβ-Cer<br>NeuAcα-8NeuAcα-3	-	-
321	GQ1b	NeuAcα-8NeuAcα-3Galβ-3GalNAcβ-4Galβ-4Glcβ-Cer<br>NeuAcα-8NeuAcα-3	-	-
322	GSC-442	GalNAcβ-4Galβ-4Glcβ-Cer36<br>NeuAcα-6	-	-
323	GSC-68	NeuAcα-6Galβ-3GalNAcβ-4Galβ-4Glcβ-Cer36	-	1,386
324	GSC-107	NeuAcα-6Galβ-3GalNAcβ-4Galβ-4Glcβ-Cer36<br>NeuAcα-6	-	42,241
325	GSC-118	NeuAcα-3Galβ-3GalNAcβ-4Galβ-4Glcβ-Cer36<br>NeuAcα-6	-	1,356
326	GalNAc-Ser	GalNAc-Ser	-	-
327	GalNAc-Thr	GalNAc-Thr	60	6
328	BSM-Di-A1-AO	NeuGcα-6GalNAc-AO	-	-
329	BSM-Di-A2-AO	NeuAcα-6GalNAc-AO	-	30,644
330	GalNAcα-3GalNAc	GalNAcα-3GalNAc-DH	211	-
331	Galß-3GalNAc	Galβ-3GalNAc-DH	-	-
332	Galß-3GalNAc-AO	Galβ-3GalNAc-AO	-	-
333	Galß-6GalNAc	Galβ-6GalNAc-DH	47	-
334	Galß-6GalNAc-AO	Galβ-6GalNAc-AO	202	-
335	Man-Ser	Manα-Ser	134	200
336	Man-Ser-Succ	Man-Ser-Succ	-	-
337	Man-Thr	Man-Thr	10	-
338	Man-Thr-Succ	Man-Thr-Succ	-	-
339	A8/1	GlcNAcα-4Galβ-OX	-	-
340	A8/2	SU-6<br>Fucα-3GlcNAcβ-OY	-	-
341	A15/1	SU-6GlcNAcβ-OY	-	-
342	A15/3	GlcNAcα-4Galβ-3Galβ-OX<br>Fucα-2	-	-
343	B13/a	GlcAβ-3Galβ-3GlcNAcβ-OX	-	-
344	Notch-1	Fucα-Thr	-	-
345	Notch-2	GlcNAcβ-3Fucα-Thr	-	-
346	Notch-3	Galβ-4GlcNAcβ-3Fucα-Thr	-	-
347	GSC-488	NeuAcα-3Galβ-3GalNAcβ-C30	-	-
348	GSC-491	NeuAcα-3Galβ-3(6-deoxy-6-carboxymethyl)GalNAcβ-C30	-	-
349	GSC-489	SU-6<br>NeuAcα-3Galβ-3GalNAcβ-C30	-	-
350	DST	NeuAcα-3Galβ-3GalNAc-DH<br>NeuAcα-6	-	1,504
351	DST-AO	NeuAcα-3Galβ-3GalNAc-AO<br>NeuAcα-6	-	709
352	GSC-490	NeuAcα-3Galβ-3GalNAcβ-C30<br>NeuAcα-6	-	-
353	GlcNAcß-3Fuc-AO	GlcNAcβ-3Fuc-AO	452	87
354	GlcNAcß1-2Fuc-AO	GlcNAcβ-2Fuc-AO	-	-
355	GlcNAcß1-4Fuc-AO	GlcNAcβ-4Fuc-AO	994	-
356	GlcNAcß-2Man-AO	GlcNAcβ-2Man-AO	-	-
357	SA2(α8)	NeuAcα-8NeuAc-DH	-	-
358	SA3(α8)	NeuAcα-8NeuAcα-8NeuAc-DH	-	-
359	SA4(α8)	NeuAcα-8NeuAcα-8NeuAcα-8NeuAc-DH	-	-
360	SA5(α8)	NeuAcα-8NeuAcα-8NeuAcα-8NeuAcα-8NeuAc-DH	-	-
361	SA6(α8)	NeuAcα-8NeuAcα-8NeuAcα-8NeuAcα-8NeuAcα-8NeuAc-DH	-	-
362	SA7(α8)	NeuAcα-8NeuAcα-8NeuAcα-8NeuAcα-8NeuAcα-8NeuAcα-8NeuAc-DH	-	-

Supporting information

363	SA8(α8)	NeuAcα-8NeuAcα-8NeuAcα-8NeuAcα-8NeuAcα-8NeuAc-8NeuAcα-8NeuAc-DH	-	-
364	SA9(α8)	NeuAcα-8NeuAcα-8NeuAcα-8NeuAcα-8NeuAcα-8NeuAc-8NeuAcα-8NeuAc-8NeuAcα-DH	-	-
365	SA10(α8)	NeuAcα-8NeuAcα-8NeuAcα-8NeuAcα-8NeuAcα-8NeuAcα-8NeuAcα-8NeuAcα-8NeuAcα-8NeuAc-DH	-	-
366	HA-S4	GlcAβ-3GlcNAcβ-4GlcAβ-3GlcNAc-DH	7	-
367	HA-S14	GlcAβ-3GlcNAcβ-4GlcAβ-3GlcNAcβ-4GlcAβ-3GlcNAcβ-4GlcAβ-3GlcNAcβ-4GlcAβ-3GlcNAcβ-4GlcAβ-3GlcNAcβ-4GlcAβ-3GlcNAc-DH	-	-
368	Hep-Di IS	ΔUA-4GlcNS-DH | | SU-2 | SU-6	-	-
369	Hep-Di-IS-AO	ΔUA-4GlcNS-AO | | SU-2 | SU-6	-	-
370	CSA-4	ΔUA-3GalNAcβ-4GlcAβ-3GalNAc-DH | | SU-4 SU-4	-	-
371	CSA-14	ΔUA-3GalNAcβ-4GlcAβ-3GalNAcβ-4GlcAβ-3GalNAcβ-4GlcAβ-3GalNAcβ-4GlcAβ-3GalNAcβ-4GlcAβ-3GalNAcβ-4GlcAβ-3GalNAc-DH 							 SU-4 SU-4 SU-4 SU-4 SU-4 SU-4 SU-4	-	-
372	CSB-4	ΔUA-3GalNAcβ-4IdoAα-3GalNAc-DH | | SU-4 SU-4	-	-
373	CSB-14	ΔUA-3GalNAcβ-4IdoAα-3GalNAcβ-4IdoAα-3GalNAcβ-4IdoAα-3GalNAcβ-4IdoAα-3GalNAcβ-4IdoAα-3GalNAcβ-4IdoAα-3GalNAc-DH 							 SU-4 SU-4 SU-4 SU-4 SU-4 SU-4 SU-4	201	4,531
374	CSC-4	ΔUA-3GalNAcβ-4GlcAβ-3GalNAc-DH | | SU-6 SU-6	-	-
375	CSC-14	ΔUA-3GalNAcβ-4GlcAβ-3GalNAcβ-4GlcAβ-3GalNAcβ-4GlcAβ-3GalNAcβ-4GlcAβ-3GalNAcβ-4GlcAβ-3GalNAcβ-4GlcAβ-3GalNAc-DH 							 SU-6 SU-6 SU-6 SU-6 SU-6 SU-6 SU-6	-	2,050
376	Hep-4-AO	SU-6 	 ΔUA-4GlcNSα-4IdoAα-4GlcNS-AO 			 SU-6 SU-2 SU-6	-	-
377	Hep-14-AO	SU-2 | ΔUA-4GlcNSα-4IdoAα-4GlcNSα-4IdoAα-4GlcNSα-4IdoAα-4GlcNSα-4IdoAα-4GlcNSα-4IdoAα-4GlcNSα-4IdoAα-4GlcNS-AO |											| SU-6 SU-2 SU-6 SU-2 SU-6 SU-2 SU-6 SU-2 SU-6 SU-2 SU-6 SU-2 SU-6	41	-
378	HS-S4-AO	GlcAβ-4GlcNAcα-4GlcAβ-4aMan-AO	-	-
379	HS-S8-AO	GlcAβ-4GlcNAcα-4GlcAβ-4GlcNAcα-4GlcAβ-4GlcNAcα-4GlcAβ-4aMan-AO	-	-
380	GN2	GlcNAcβ-4GlcNAc-DH	35	-
381	GN2.AO	GlcNAcβ-4GlcNAc-AO	274	-
382	GN3	GlcNAcβ-4GlcNAcβ-4GlcNAc-DH	-	-
383	GN3.AO	GlcNAcβ-4GlcNAcβ-4GlcNAc-AO	-	-
384	GN4.AO	GlcNAcβ-4GlcNAcβ-4GlcNAcβ-4GlcNAc-AO	-	-
385	GN5.AO	GlcNAcβ-4GlcNAcβ-4GlcNAcβ-4GlcNAcβ-4GlcNAc-AO	-	-
386	GN6.AO	GlcNAcβ-4GlcNAcβ-4GlcNAcβ-4GlcNAcβ-4GlcNAcβ-4GlcNAc-AO	-	-
387	GN7.AO	GlcNAcβ-4GlcNAcβ-4GlcNAcβ-4GlcNAcβ-4GlcNAcβ-4GlcNAcβ-4GlcNAc-AO	-	-
388	GN8.AO	GlcNAcβ-4GlcNAcβ-4GlcNAcβ-4GlcNAcβ-4GlcNAcβ-4GlcNAcβ-4GlcNAcβ-4GlcNAc-AO	-	-
389	Man4(ß4)	Manβ-4Manβ-4Manβ-4Man-DH	37	-
390	Man5(ß4)	Manβ-4Manβ-4Manβ-4Manβ-4Man-DH	-	-
391	Man6(ß4)	Manβ-4Manβ-4Manβ-4Manβ-4Manβ-4Man-DH	55	-
392	Xyl5(ß4)	Xylβ-4Xylβ-4Xylβ-4Xylβ-4Xyl-DH	-	-
393	Xyl6(ß4)	Xylβ-4Xylβ-4Xylβ-4Xylβ-4Xylβ-4Xyl-DH	202	97
394	Ara6(α5)	Araα-5Araα-5Araα-5Araα-5Araα-5Ara-DH	-	-
395	Ara7(α5)	Araα-5Araα-5Araα-5Araα-5Araα-5Araα-5Ara-DH	23	-
396	Glc2(α2)-AO	Glcα-2Glc-AO	-	-
397	Glc2(α3)-AO	Glcα-3Glc-AO	629	66
398	Malto-2-AO	Glcα-4Glc-AO	209	-
399	Malto-3-AO	Glcα-4Glcα-4Glc-AO	456	-
400	Malto-4-AO	Glcα-4Glcα-4Glcα-4Glc-AO	79	-
401	Malto-5-AO	Glcα-4Glcα-4Glcα-4Glcα-4Glc-AO	517	202
402	Malto-6-AO	Glcα-4Glcα-4Glcα-4Glcα-4Glcα-4Glc-AO	561	-
403	Malto-7-AO	Glcα-4Glcα-4Glcα-4Glcα-4Glcα-4Glcα-4Glc-AO	521	-
404	Malto-8-AO	Glcα-4Glcα-4Glcα-4Glcα-4Glcα-4Glcα-4Glcα-4Glc-AO	709	-
405	Malto-9-AO	Glcα-4Glcα-4Glcα-4Glcα-4Glcα-4Glcα-4Glcα-4Glcα-4Glc-AO	253	107
406	Malto-10-AO	Glcα-4Glcα-4Glcα-4Glcα-4Glcα-4Glcα-4Glcα-4Glcα-4Glcα-4Glc-AO	158	-
407	Malto-11-AO	Glcα-4Glcα-4Glcα-4Glcα-4Glcα-4Glcα-4Glcα-4Glcα-4Glcα-4Glcα-4Glc-AO	327	-
408	Malto-12-AO	Glcα-4Glcα-4Glcα-4Glcα-4Glcα-4Glcα-4Glcα-4Glcα-4Glcα-4Glcα-4Glcα-4Glc-AO	518	-
409	Malto-13-AO	Glcα-4Glcα-4Glcα-4Glcα-4Glcα-4Glcα-4Glcα-4Glcα-4Glcα-4Glcα-4Glcα-4Glcα-4Glc-AO	461	-
410	Dextran-2-AO	Glcα-6Glc-AO	605	-

411	Dextran-3-AO	Glcα-6Glcα-6Glc-AO	115	-
412	Dextran-4-AO	Glcα-6Glcα-6Glcα-6Glc-AO	-	-
413	Dextran-5-AO	Glcα-6Glcα-6Glcα-6Glcα-6Glc-AO	56	-
414	Dextran-6-AO	Glcα-6Glcα-6Glcα-6Glcα-6Glcα-6Glc-AO	-	-
415	Dextran-7-AO	Glcα-6Glcα-6Glcα-6Glcα-6Glcα-6Glcα-6Glc-AO	-	-
416	Dextran-8-AO	Glcα-6Glcα-6Glcα-6Glcα-6Glcα-6Glcα-6Glcα-6Glc-AO	-	-
417	Dextran-9-AO	Glcα-6Glcα-6Glcα-6Glcα-6Glcα-6Glcα-6Glcα-6Glcα-6Glc-AO	-	-
418	Dextran-10-AO	Glcα-6Glcα-6Glcα-6Glcα-6Glcα-6Glcα-6Glcα-6Glcα-6Glcα-6Glc-AO	-	-
419	Dextran-11-AO	Glcα-6Glcα-6Glcα-6Glcα-6Glcα-6Glcα-6Glcα-6Glcα-6Glcα-6Glcα-6Glc-AO	-	-
420	Dextran-12-AO	Glcα-6Glcα-6Glcα-6Glcα-6Glcα-6Glcα-6Glcα-6Glcα-6Glcα-6Glcα-6Glcα-6Glc-AO	-	-
421	Dextran-13-AO	Glcα-6Glcα-6Glcα-6Glcα-6Glcα-6Glcα-6Glcα-6Glcα-6Glcα-6Glcα-6Glcα-6Glcα-6Glc-AO	-	-
422	Lam-2-AO	Glcβ-3Glc-AO	508	-
423	Lam-3-AO	Glcβ-3Glcβ-3Glc-AO	227	-
424	Lam-4-AO	Glcβ-3Glcβ-3Glcβ-3Glc-AO	-	-
425	Lam-5-AO	Glcβ-3Glcβ-3Glcβ-3Glcβ-3Glc-AO	-	-
426	Lam-6-AO	Glcβ-3Glcβ-3Glcβ-3Glcβ-3Glcβ-3Glc-AO	-	-
427	Lam-7-AO	Glcβ-3Glcβ-3Glcβ-3Glcβ-3Glcβ-3Glcβ-3Glc-AO	-	-
428	Curd-8-AO	Glcβ-3Glcβ-3Glcβ-3Glcβ-3Glcβ-3Glcβ-3Glcβ-3Glc-AO	-	-
429	Curd-9-AO	Glcβ-3Glcβ-3Glcβ-3Glcβ-3Glcβ-3Glcβ-3Glcβ-3Glcβ-3Glc-AO	-	-
430	Curd-10-AO	Glcβ-3Glcβ-3Glcβ-3Glcβ-3Glcβ-3Glcβ-3Glcβ-3Glcβ-3Glcβ-3Glc-AO	-	-
431	Curd-11-AO	Glcβ-3Glcβ-3Glcβ-3Glcβ-3Glcβ-3Glcβ-3Glcβ-3Glcβ-3Glcβ-3Glcβ-3Glc-AO	-	-
432	Curd-12-AO	Glcβ-3Glcβ-3Glcβ-3Glcβ-3Glcβ-3Glcβ-3Glcβ-3Glcβ-3Glcβ-3Glcβ-3Glcβ-3Glc-AO	-	-
433	Curd-13-AO	Glcβ-3Glcβ-3Glcβ-3Glcβ-3Glcβ-3Glcβ-3Glcβ-3Glcβ-3Glcβ-3Glcβ-3Glcβ-3Glcβ-3Glc-AO	54	-
434	Cello-2-AO	Glcβ-4Glc-AO	357	-
435	Cello-3-AO	Glcβ-4Glcβ-4Glc-AO	-	-
436	Cello-4-AO	Glcβ-4Glcβ-4Glcβ-4Glc-AO	-	-
437	Cello-5-AO	Glcβ-4Glcβ-4Glcβ-4Glcβ-4Glc-AO	47	-
438	Cello-6-AO	Glcβ-4Glcβ-4Glcβ-4Glcβ-4Glcβ-4Glc-AO	-	-
439	Cello-8-AO	Glcβ-4Glcβ-4Glcβ-4Glcβ-4Glcβ-4Glcβ-4Glcβ-4Glc-AO	-	-
440	Cello-9-AO	Glcβ-4Glcβ-4Glcβ-4Glcβ-4Glcβ-4Glcβ-4Glcβ-4Glcβ-4Glc-AO	-	-
441	Cello-10-AO	Glcβ-4Glcβ-4Glcβ-4Glcβ-4Glcβ-4Glcβ-4Glcβ-4Glcβ-4Glcβ-4Glc-AO	-	-
442	Cello-11-AO	Glcβ-4Glcβ-4Glcβ-4Glcβ-4Glcβ-4Glcβ-4Glcβ-4Glcβ-4Glcβ-4Glcβ-4Glc-AO	-	-
443	Cello-12-AO	Glcβ-4Glcβ-4Glcβ-4Glcβ-4Glcβ-4Glcβ-4Glcβ-4Glcβ-4Glcβ-4Glcβ-4Glcβ-4Glc-AO	-	-
444	Cello-13-AO	Glcβ-4Glcβ-4Glcβ-4Glcβ-4Glcβ-4Glcβ-4Glcβ-4Glcβ-4Glcβ-4Glcβ-4Glcβ-4Glcβ-4Glc-AO	6	-
445	Pust-2-AO	Glcβ-6Glc-AO	297	-
446	Pust-3-AO	Glcβ-6Glcβ-6Glc-AO	32	-
447	Pust-4-AO	Glcβ-6Glcβ-6Glcβ-6Glc-AO	-	-
448	Pust-5-AO	Glcβ-6Glcβ-6Glcβ-6Glcβ-6Glc-AO	128	-
449	Pust-6-AO	Glcβ-6Glcβ-6Glcβ-6Glcβ-6Glcβ-6Glc-AO	-	-
450	Pust-7-AO	Glcβ-6Glcβ-6Glcβ-6Glcβ-6Glcβ-6Glcβ-6Glc-AO	-	-
451	Pust-8-AO	Glcβ-6Glcβ-6Glcβ-6Glcβ-6Glcβ-6Glcβ-6Glcβ-6Glc-AO	-	-
452	Pust-9-AO	Glcβ-6Glcβ-6Glcβ-6Glcβ-6Glcβ-6Glcβ-6Glcβ-6Glcβ-6Glc-AO	-	-
453	Glc2ß(2).AO	Glcβ-2Glc-AO	544	-
454	Gal	Gal-DH	200	52
455	Gal-AO	Gal-AO	470	-
456	GalNAc	GalNAc-DH	328	145
457	GalNAc-AO	GalNAc-AO	167	-
458	Glc	Glc-DH	104	61
459	Glc-AO	Glc-AO	122	-
460	GN	GlcNAc-DH	-	-
461	GN-AO	GlcNAc-AO	282	-
462	Man	Man-DH	490	-
463	Man-AO	Man-AO	172	246
464	Fuc	Fuc-DH	117	44
465	Fuc-AO	Fuc-AO	236	-
466	NeuAc	NeuAc-DH	175	-
467	NeuAc-AO	NeuAc-AO	-	-
468	NeuGc	NeuGc-DH	-	-
469	NeuGc-AO	NeuGc-AO	-	-
470	Rha	Rha-DH	-	-
471	Rha-AO	Rha-AO	815	-

Supporting information

472	Galα-6Glc-AO	Galα-6Glc-AO	88	-
473	(6P)-Glc-AO	P-6Glc-AO	-	-
474	(6P)-Man	P-6Man-DH	5	-
475	(6P)-Man-AO	P-6Man-AO	-	-
476	(6P)-Man5	P-6Manα-3Manα-3Manα-3Manα-2Man-DH	-	-
477	(6P)-Fructose-AO	P-6Fru-AO	120	-
478	SU-Tyr	SU-Tyr	26	-
479	SU-Cholesterol	SU-Cholesterol	-	-
480	GN-Asn	GlcNAc-Asn	300	-
481	Xyl3Glc4	Xylα-6<br>| Glcβ-4Glcβ-4Glcβ-4Glc-DH<br>| | Xylα-6 Xylα-6	-	-
482	GSC-284	GalNAcβ-6Galβ-4Glcβ-Cer36<br>| NeuAcα-3	-	-
483	GSC-575	GalNAcβ-4Galβ-3Galβ-C30<br>| NeuAcα-3	-	-
484	GSC-70	NeuAcα-6Galβ-6GalNAcβ-4Galβ-4Glcβ-Cer36	-	-
485	GSC-154	NeuAcα-3Galβ-4GlcNAcβ-6Galβ-4Glcβ-Cer36<br>| Fucα-3	63	7
486	GSC-446	NeuAcα-3Galβ-4GlcNAcβ-6GalNAcα-3Galβ-4Glc-C30	-	16,602
487	GSC-441	NeuAcα-3Galβ-4GlcNAcβ-6GalNAcα-3Galβ-4Glcβ-C30	-	11,817
488	GSC-384	NeuAcα-3Galβ-4GlcNAcβ-4GalNAcβ-3Galβ-4Glcβ-C30<br>| Fucα-3	-	141
489	Glc(α6,α4,α4)	Glcα-6Glcα-4Glcα-4Glc-DH	-	-
490	Glc(α6,α4,α4)-AO	Glcα-6Glcα-4Glcα-4Glc-AO	117	-
491	O1.AO	GlcNAcβ-3<br>| Gal-AO<br>| GlcNAcβ-6	-	-
492	Rutinose.AO	Rhaα-6Glc-AO	541	-

-, less than 1.
*, Average fluorescence intensity of the duplicate spots.
**, Major component.

The oligosaccharide probes are all lipid-linked. Unless otherwise indicated they are NGLs prepared from reducing oligosaccharides by reductive amination with the amino lipid, 1,2-dihexadecyl-*sn*-glycero-3-phosphoethanolamine (DHPE). AO, NGLs prepared from reducing oligosaccharides by oxime ligation with an aminooxy (AO) functionalized DHPE [1]; Cer, natural glycolipids with various ceramide moieties; Cer36, synthetic glycolipids with ceramide having a total of 36 carbon atoms; C30, a synthetic lipid [2-(tetradecyl)hexadecanol] with 30 carbon atoms. OX and OY designate, respectively, the C1-4 fragment and the C5-6 fragments of GalNAcol of reduced oligosaccharides after mild periodate oxidation followed by reductive amination with DHPE [2]. ∆UA, 4,5-unsaturated hexuronic acid; aMan, 2,5-anhydro-mannose; aGal, 3,6-anhydro-galactose.

1. Liu Y, Feizi T, Campanero-Rhodes MA, Childs RA, Zhang Y, Mulloy B, et al. Neoglycolipid Probes Prepared via Oxime Ligation for Microarray Analysis of Oligosaccharide-Protein Interactions. Chemistry & Biology. 2007;14(7):847-59. doi: 10.1016/j.chembiol.2007.06.009.
2. Chai W, Stoll MS, Galustian C, Lawson AM, Feizi T. Neoglycolipid technology: deciphering information content of glycome. Methods Enzymol. 2003;362:160-95.
