# Supplementary material for: Extended receptor repertoire of an adenovirus associated with human obesity
Source: PLoS Pathog. 2025 Jan 30;21(1):e1012892. doi: 10.1371/journal.ppat.1012892 (PMC11813153; doi:10.1371/journal.ppat.1012892)
Supplement: S2 Table — (PDF) [file ppat.1012892.s016.pdf]

S2 Table. Excerpt from the main Table S1 of the fluorescence signals obtained for large arrays of HAdV-D36 and HAdV-D37.

Glycan No.	Probe	Structure	HAdV-D36	HAdV-D37
44	NeuAcα-(3')Lac	NeuAcα-3Galβ-4Glc-DH	-	50,438
45	NeuAcα-(3')Lac-AO	NeuAcα-3Galβ-4Glc-AO	-	16,272
46	Neu4,5Ac-(3')Lac	(4-OAc)NeuAcα-3Galβ-4Glc-DH	7,279	12,366
47	Neu4,5Ac-(3')Lac-AO	(4-OAc)NeuAcα-3Galβ-4Glc-AO	107	297
107	Neu5,9Ac-(6')LN	(9-OAc)NeuAcα-6Galβ-4GlcNAc-DH	-	-
114	GSC-513	(9-OAc)NeuAcα-3Galβ-3GlcNAcβ-C30<br>| Fucα-4	-	-
115	GSC-511	(9-OAc)NeuAcα-3Galβ-4GlcNAcβ-C30<br>| Fucα-3	-	-
311	GD1a-hexa	NeuAcα-3Galβ-3GalNAcβ-4Galβ-4Glc-DH<br>| NeuAcα-3	-	15,425
