# Supplementary material for: Extended receptor repertoire of an adenovirus associated with human obesity
Source: PLoS Pathog. 2025 Jan 30;21(1):e1012892. doi: 10.1371/journal.ppat.1012892 (PMC11813153; doi:10.1371/journal.ppat.1012892)
Supplement: S3 Table — (PDF) [file ppat.1012892.s017.pdf]

Supporting information

S3 Table. Data collection and refinement statistics of selected datasets. All other structures are listed in [1, 2].

	HAdV-D36 FK + 4-O-Ac-3'SL	HAdV-D36 FK + 2-O-me-Neu4,5Ac2	HAdV-D36 FK + 2-O-me-Neu4,5,9Ac3	HAdV-D37 FK + 2-O-me-Neu4,5Ac2
Data Collection				
Beamline	SLS, X06DA	SLS, X06DA	SLS, X06DA	SLS, X06DA
Space Group	$P2_12_12_1$	$P2_12_12_1$	$P2_12_12_1$	$P2_1$
Cell Dimensions				
a, b, c [Å]	51.13, 98.64, 99.83	59.46, 99.16, 110.99	59.59, 99.21, 111.04	56.88, 66.73, 73.95
α, β, γ [°]	90, 90, 90	90, 90, 90	90,90,90	90, 97.75, 90
Resolution [Å]	50 - 1.57 (1.67 - 1.57)	50 - 1.76 (1.85 - 1.76)	50 – 1.90 (2.00 – 1.90)	50 - 1.65 (1.74 - 1.65)
$CC_{1/2}$ [%]	99.9 (65.1)	99.9 (64.7)	99.7 (53.2)	99.9 (49.5)
R_{meas} [%]	9.8 (103.2)	14.4 (181.7)	23.1 (211.1)	7.0 (157.5)
I/σ(I)	14.0 (1.84)	14.5 (1.53)	10.4 (1.35)	15.8 (1.22)
Completeness [%]	98.8 (94.3)	99.0 (94.1)	99.4 (96.4)	99.7 (98.7)
Redundancy	6.8 (6.3)	13.1 (12.4)	13.4 (13.0)	6.8 (6.9)
Wilson B-Factor [$Å^2$]	25.4	31.3	33.1	34.6
Refinement				
Resolution [Å]	45.50 - 1.57	48.43 - 1.69	48.45 – 1.90	73.27 - 1.65
No. of Reflections	133755	65546	52253	65451
R_{work} / R_{free} [%]	15.51 / 18.70	16.50 / 18.39	16.91 / 20.62	15.77 / 19.03
No. of Atoms				
Protein	4396	4351	4298	4242
Solvent	463	439	309	310
Carbohydrate	83	75	140	75
B-Factors [$Å^2$]				
Protein	19.7	30.8	32.2	34.1
Solvent	29.8	41.6	40.9	43.1
Carbohydrate	28.0	26.8	33.7	35.6
R. m. s. deviations				
Bond Length [Å]	0.012	0.007	0.011	0.010
Bond Angle [°]	1.240	0.894	1.124	1.056
Ramachandran Plot				
Favored	96.6 %	96.9%	97.0 %	96.6 %
Allowed	3.4 %	3.1%	3.0 %	3.4 %
Disallowed	0.0 %	0.0%	0.0 %	0.0 %

Supporting information

1. Bachmann P. Structural Insights into the Sialic Acid Binding Modes of Human Adenovirus D-36 and D-37 Fiber Knob Domains Tübingen: University of Tübingen; 2016.
2. Pfenning V. Structural Investigations of the Human Adenovirus 36 Fiber Knob and its Candidate Receptors. Tübingen: University of Tübingen; 2014.
