# Supplementary material for: Extended receptor repertoire of an adenovirus associated with human obesity
Source: PLoS Pathog. 2025 Jan 30;21(1):e1012892. doi: 10.1371/journal.ppat.1012892 (PMC11813153; doi:10.1371/journal.ppat.1012892)
Supplement: S4 Table — (PDF) [file ppat.1012892.s018.pdf]

Supporting information

S4 Table. Supplementary glycan microarray document based on MIRAGE Glycan Microarray guidelines (doi:10.3762/mirage.3) for the glycan array experiments.

**Classification**	**Guidelines**
**1. Sample: Glycan Binding Sample**	
Description of Sample	Sample name:<br>His-tagged fiber knob (FK) proteins of HAdV-D36 and HAdV-D37<br>Origin: recombinant<br>Method of preparation:<br>Please see the *Materials and Methods* section in the main text.
Sample modifications	Not relevant.
Assay protocol	Microarray analyses were performed essentially as described [1], for modifications of the protocols please see "Glycan array screening" section under the *Materials and Methods* section in the main text.
**2. Glycan Library**	
Glycan description for defined glycans	Two glycan microarrays were used:<br>1) A broad-spectrum screening glycan microarray containing 492 sequence-defined lipid-linked glycan probes was used as previously described [2]. The probe names and corresponding structures are in **Table S1** (in-house designation 'Array Sets 32-39').<br>2) A focused sialyl glycan array in dose-response format containing 8 neoglycolipid (NGL) probes (see **Figure 4B**).<br>The glycan probes are from the collection assembled in the course of research in the Glycosciences Laboratory https://glycosciences.med.ic.ac.uk/glycanLibraryList.html.
Glycan description for undefined glycans	Not relevant.
Glycan modifications	For NGLs, unless otherwise specified these were prepared from reducing oligosaccharides by reductive amination with the amino lipid, 1,2-dihexadecyl-*sn*-glycero-3-phosphoethanolamine [(DHPE) [3]]; AO, NGLs prepared from reducing oligosaccharides by oxime ligation with an aminooxy functionalized DHPE [(AOPE) [4]].<br>For full description on the definition of lipid moieties of the glycan probes please see https://glycosciences.med.ic.ac.uk/docs/lipids.pdf.
**3. Printing Surface; e.g., Microarray Slide**	
Description of surface	Nitrocellulose-coated glass microarray slides.
Manufacturer	In the original microarray study, 16-pad Nexterion Slides NC from Schott (Elmsford, USA) were u tilized. Currently, similar products are available, such as the 16-pad UniSart 3D Microarray Slides from Sartorius (Goettingen, Germany).

Custom preparation of surface	Not relevant.
Non-covalent Immobilisation	For the broad-spectrum screening glycan microarray (492 probes), the lipid-linked oligosaccharide probes were formulated as liposomes by adding carrier lipids, phosphatidylcholine (from egg yolk) and cholesterol (both from SIGMA, >90%) for arraying and non-covalent immobilization on nitrocellulose-coated glass slides [1].<br>For the dose-response sialyl glycan array (8 probes), 1,2-dihexanoyl-*sn*-glycero-3-phosphocholine (DHPC) instead of phosphatidylcholine was used in the liposome formulation.
**4. Arrayer (Printer)**	
Description of Arrayer	Piezorray instrument (PerkinElmer LAS, Beaconsfield, UK)
Dispensing mechanism	Non-contact liquid delivery with four dispensing tips.
Glycan deposition	Approximately 0.33 nl was printed per spot.<br>Lipid-linked glycan probes were printed at 2 and 5 fmol per spot.
Printing conditions	The printing solutions were all aqueous based containing Cy3 NHS ester (GE Healthcare) at 20 ng/ml (26 fmol/µl) as a marker to monitor the printing process. Printing was performed at ambient temperature and relative humidity of 58%.<br>The 'liposome' printing solutions contained 100 pmol/µl of phosphatidylcholine (or DHPC) and cholesterol as lipid carriers in addition to the lipid-linked glycan probes. The concentrations of the lipid-linked glycan probes used in the broad-spectrum screening glycan microarray were 5 pmol/µl and 15 pmol/µl, corresponding to 2 fmol and 5 fmol per spot, respectively. For the dose-response sialyl glycan array, the probe concentrations were 1 pmol/µl, 2.5 pmol/µl, 5 pmol/µl, and 15 pmol/µl, corresponding to spot levels of 0.3 fmol, 0.8 fmol, 1.7 fmol, and 5 fmol, respectively.
**5. Glycan Microarray with "Map"**	
Array layout	The 492 lipid-linked probes in the broad-spectrum screening arrays were printed on multiple subarrays for parallel binding analyses. Each array slide contained 16-pad subarrays. Each pad was set up for printing 64 probes maximum, each at 2 levels in duplicate (four spots for one probe in a row); up to 256 spots (16x16) in total in each pad.<br>The dose-response focused sialyl glycan array set containing 8 NGL probes in dose-response format, each at 4 levels in duplicate as mentioned above.
Glycan identification and quality control	The quality control of the glycan microarrays was routinely carried out with (i) a panel of biotinylated plant lectins (Vector Laboratories), e.g. *Ricinus Communis* Agglutinin I (RCA_{120}), *Aleuria aurantia* lectin (AAL), Concanavalin A (ConA) and wheat germ agglutinin (WGA), and (ii) anti-carbohydrate antibodies. Some of the resulting data have been published [2], and others will be shared through the GlyGen Glycan Array Repository, which is currently under development as part of the NIH-funded GlyGen initiative (https://www.glygen.org/) and has now entered its final testing phase.
**6. Detector and Data Processing**	
Scanning hardware	GenePix 4300A (Molecular Devices, UK)
Scanner settings	Scanning resolution: 10 µm / pixel<br>Laser channel: Red (scan wavelength 635 nm)

	PMT: 350<br>Scan power: 100% to achieve maximum signal without spot saturation.
Image analysis software	GenePix Pro 7 (Molecular Devices)
Data processing	The gpr files were entered into an in-house microarray database using software (designed by Mark Stoll, http://www.beilstein-institut.de/en/publications/proceedings/glyco-2009) for data processing. No particular normalization method or statistical analysis was used for the results of the screening arrays.
**7. Glycan Microarray Data Presentation**	
Data presentation	The full results of the broad-spectrum screening arrays are in Figure 4A and Table S1. The binding results focused on sialyl glycan probes in the dose-response array set are in Figure 4B.
**8. Interpretation and Conclusion from Microarray Data**	
Data interpretation	No software or algorithms were used to interpret processed data.
Conclusions	Under the assay conditions used, the two recombinant FK proteins showed differing binding profiles to sialyl glycans in the microarrays. HAdV-D36 FK bound exclusively to 4-O-Ac-3'SL with Neu4,5Ac_2 sialic acid residue, in contrast to HAdV-D37 FK which bound to a broader range of sialyl glycan probes with a preference to Sialyl 2-3 linkage and unmodified sialic acid Neu5Ac.

1. Liu Y, Childs RA, Palma AS, Campanero-Rhodes MA, Stoll MS, Chai W, et al. Neoglycolipid-Based Oligosaccharide Microarray System: Preparation of NGLs and Their Noncovalent Immobilization on Nitrocellulose-Coated Glass Slides for Microarray Analyses. Carbohydrate Microarrays. Methods in Molecular Biology2012. p. 117-36.
2. Palma AS, Liu Y, Childs RA, Herbert C, Wang D, Chai W, et al. The human epithelial carcinoma antigen recognized by monoclonal antibody AE3 is expressed on a sulfoglycolipid in addition to neoplastic mucins. Biochemical and Biophysical Research Communications. 2011;408(4):548-52. doi: 10.1016/j.bbrc.2011.04.055.
3. Chai W, Stoll MS, Galustian C, Lawson AM, Feizi T. Neoglycolipid Technology: Deciphering Information Content of Glycome. Recognition of Carbohydrates in Biological Systems, Part A: General Procedures. Methods in Enzymology2003. p. 160-95.
4. Liu Y, Feizi T, Campanero-Rhodes MA, Childs RA, Zhang Y, Mulloy B, et al. Neoglycolipid Probes Prepared via Oxime Ligation for Microarray Analysis of Oligosaccharide-Protein Interactions. Chemistry & Biology. 2007;14(7):847-59. doi: 10.1016/j.chembiol.2007.06.009.
