# Supplementary material for: Extended receptor repertoire of an adenovirus associated with human obesity
Source: PLoS Pathog. 2025 Jan 30;21(1):e1012892. doi: 10.1371/journal.ppat.1012892 (PMC11813153; doi:10.1371/journal.ppat.1012892)
Supplement: S1 Text — (PDF) [file ppat.1012892.s019.pdf]

Extended materials and methods

Virus production and labeling. Confluent A549 cells were split in 1:2 in 175 cm^2 cell culture flasks (Sarstedt AG & Co. KG) and incubated overnight in 37°C. The next day, the growth medium [DPH (Dulbecco's Modified Eagle Medium (DMEM) containing $NaHCO_3$ (0.7 g/L), 20 mM HEPES pH 7.4 and 0.1 % PenStrep (10 U/mL penicillin + 10 µg/mL streptomycin (Gibco))] supplemented with 5 % fetal bovine serum (FBS, Thermo Scientific) was removed and 5 mL of DPH supplemented with 1 % FBS) was added to each flask. The inoculation material (i.m.; previously prepared from infected A549 cells) was freeze-thawed three times in 37°C and -80°C, and 100 µL were added to each flask and incubated for 90 mins. The medium with i.m. was poured off to remove unbound virions and 30 mL of DPH supplemented with 1 % FBS was added to each flask. About 72 h post infection, the cells were harvested, and the pellet was re-suspended in 6 ml of DPH. After three cycles of freeze-thaw,6 ml of 1,1,1,2,3,4,4,5,5,5- Decafluoropentane (Sigma-Aldrich, St Louis, USA) was added to the cell suspension and shaken vigorously by hand for 2-3 min. After centrifugation for 5 min at 3000 rpm, the top layer containing the virus particles was added onto a CsCl gradient with densities 1.27 g/mL, 1.32 g/mL and 1.37 g/mL and ultracentrifuged (SW41Ti rotor, OptimaTM L-80 XP Ultracentrifuge, Beckman Coulter) at 25 000 rpm, 4°C for 90 mins. The lower band, which contained the virus particles, was harvested and desalted on a NAP column (GE Healthcare, Buckinghamshire, UK) in sterile PBS. The virion concentration was measured with NanoDrop (ND-1000 Spectrophotometer, Saveen Werner), and desired quantity of virions were aliquoted to be labelled further. The remaining virions was mixed with glycerol to a final concentration of 10 %, aliquoted and stored in -80°C until further use. Production of ^{35}S-radiolabelled virions was performed as described previously [1], with the following exceptions: After 24 h of infection, cells were washed twice with sterile phosphate buffered saline (PBS) containing 0.05 % EDTA to get rid of traces of L-cysteine and L-methionine and starved for 2 h in DMEM without L-cysteine and L-methionine (Gibco) supplemented with 1 % FBS (Thermo Scientific), 20 mM HEPES (Fischer Scientific), 20 U/mL penicillin + 20 µg/mL streptomycin (GE Healthcare) and 4 mM L-glutamine (Gibco). The isotope ^{35}S (Easy tag express protein labeling mix, Perkin Elmer, Boston, USA) was added thereafter at a concentration of 1 mCi/flask. L-cysteine (Sigma-Aldrich) and L-methionine (Sigma-Aldrich) was added to a final concentration of 2 mM at 1 h or 4.5 h after addition of ^{35}S, respectively. L-cysteine (Sigma-Aldrich) and L-methionine (Sigma-Aldrich) were added again to a final concentration of 2 mM 24 h after addition of ^{35}S. Fluorophore labelling was performed by adding a ten-fold molar excess of Alexa Flour 488 N-hydroxysuccinimide (NHS) ester (Invitrogen) was added to freshly produced HAdV-D36 virions while vortexing the tube gently, keeping final virion concentration 1 µg/µL. The labelling mixture was wrapped with aluminium foil and incubated for 1 h at room temperature on a rotator. The labelled virions were purified by loading onto a CsCl gradient with densities 1.27 g/mL, 1.32 g/mL and 1.37 g/mL and ultracentrifuged (SW60Ti rotor, OptimaTM L-80 XP Ultracentrifuge, Beckman Coulter) at 25 000 rpm, 4°C for 2 h. The lower band was collected and desalted on a NAP column (Cytiva) in sterile PBS. Glycerol was added to a final concentration of 4 %, the virion concentration was measured with NanoDrop (ND-1000 Spectrophotometer, Saveen Werner), and the virion solution was aliquoted and stored in -80°C until further use.

Cell-binding assays. Cells were split one day before the experiment. On the day of the experiment, cells were detached with PBS containing 0.05 % EDTA and reactivated for 1 h at 37°C in growth media. Cells ($1x10^5$ cells/well) were placed on a V-shaped bottom 96-well plate and washed once with binding buffer (BB: DMEM (Gibco) supplemented with 20 mM HEPES (Fischer Scientific), 20 U/mL penicillin + 20 µg/mL streptomycin (GE Healthcare) and 1 % bovine serum albumin (Roche, Mannheim, Germany)). ^{35}S-labelled viruses (HAdV-D36: $1x10^9$ virions/well, HAdV-C5: $1x10^9$ virions/well and HAdV-D37: $5x10^8$ virions/well) diluted in BB were added to the cells and incubated at 4°C for 1 h. Cells were then pelleted and washed twice with PBS to remove unbound virions. Samples were analyzed by measuring radioactivity in a scintillation counter (1450 Microbeta, Wallac). The assays were performed with the following variations: Binding of HAdV-D36 was analysed to (i) A549, 3T3-L1, SGBS, EKVX, HEK293, FHS, HEP-G2, CHO-K1, CHO-mock, CHO-CAR, CHO-CD46, CHO-CD21, CHO-ICAM-1, CHO-CD55, CHO-Pro5, CHO-Lec2, CHO-2241 and CHO-2242 cells (ii) A549 cells pretreated for 1 h at 37°C with different concentrations of

enzymes ((Ficin, proteinase K, Bromelain, V8 protease, heparinase III (10 mU/µL, *F. heparinum*) and neuraminidase (20 mU/mL, *V. cholerae*) and inhibitors (Benzyl-GalNAc, an inhibitor of de novo *O*-glycosylation, tunicamycin (an inhibitor of N-glycosylation), and P4 (DL-threo-1-phenyl-2-palmitoylamino-3-pyrrolidino-1-propanol, an inhibitor of glycolipid synthesis). In some experiments, the binding of HAdV-D36 virus, pre-incubated 1 h at 4°C with different concentrations of SA (N-acetylneuraminic acid, Dextra Laboratories, Reading, UK), heparin (from porcine intestinal mucosa, Sigma-Aldrich) and compound 17a [2], to A549 cells was analyzed. The data represent values from three individual experiments with duplicate samples in each experiment. Error bars represent mean ± SD. Untreated cells or absence of compounds were used as controls. In case of binding assay involving fluorophore labelled virus, the assay was performed as described above. Briefly, the AF488-labelled HAdV-D36 virions were diluted in chilled binding buffer BB and compounds [Neu5Ac and Neu4,5Ac$_2$ (For functional assay, we used in-house synthesized 2-O-methyl analogue of 4-O-Neu5Ac$_2$ i.e., 2-O-me-Neu4,5Ac$_2$)] were reconstituted in DPH and placed in 4°C until used. Reactivated A549 cells (1x10^5 cells/well), plated in V-bottomed 96- well plate and washed with BB, were gently mixed with 50 µL of fluorophore-labelled virions (1x10^9 virions/well) and incubated for 1 h on ice on a rocking table. Virions, before adding to cells, were incubated with increasing concentrations (0 mM, 0.4 mM, 2 mM, 10 mM and 50 mM) of Neu5Ac, and Neu4,5Ac$_2$ on ice for 1 hr. After washing the cells with chilled PBS twice, cells were analysed by flow cytometry. The data represents values from one experiment performed with triplicate samples.

Site-directed mutagenesis using a modified Strategene protocol. The YGT and VSN mutants were prepared by site-directed mutagenesis using a modified Strategene protocol. PCR was performed in 22 cycles, routinely using annealing temperatures from 55-57°C in eight 50 µL setups and the ExactRun Polymerase (Genaxxon). Each setup contained 5 ng of template plasmid. All eight setups were pooled, incubated with 3µL DpnI (Thermo Fischer Scientific) and subjected to ethanol precipitation for 1-3 days and resuspended in in H_2O prior to transformation.

Purification of 4-*O*-acetyl-3'-sialyllactose (4-*O*-Ac-3'SL) from echidna milk oligosaccharides (EMOs). 4-*O*-Ac-3'SL was isolated from an oligosaccharide mixture derived from the milk of the Australian short-beaked echidna. EMOs [3] received from Professor Tadasu Urashima (Obihiro University) were fractionated by gel filtration chromatography on a Bio-Gel P4 column (16 x 90 cm) with elution by ammonium acetate (0.2 M) and detection by refractive index (**S14A Fig**). As detected by hexose assay, fractions 5-10 contain carbohydrate materials. The pooled fraction F6 was further fractionated by normal phase HPLC (Amide column) with elution by a gradient of H_2O/ACN containing 0.05 mM phosphate and detection at UV 196 nm (**S14B Fig**) and the subfraction 2 (F6-2) was analyzed by negative-ion electrospray mass spectrometry. The spectrum (**S14D Fig**) indicated that F6-2 contained the trisaccharide of interest as the major component ([M-H]- at m/z 674) but also contain a minor component disialylated hexasaccharide ([M-2H]2- at m/z 664 and [M-H]- at m/z 1330). Further HPLC purification using the same system but with an amine column resolved the two components (**S14C Fig**) and the mass spectra identified F6-2a as the sialylated lactose with acetyl on the 4-*O*-position of NeuAc (**S14E and S14G Fig**) and F6-2d as the disialylated hexasaccharide containing a single *O*Ac (**S14F Fig**). Quantitation was carried out by microscale orcinol assay as essentially as described [4].

References

1. Johansson SM, Nilsson EC, Elofsson M, Ahlskog N, Kihlberg J, Arnberg N. Multivalent sialic acid conjugates inhibit adenovirus type 37 from binding to and infecting human corneal epithelial cells. Antiviral Res 2007;73:92-100.
2. Caraballo R, Saleeb M, Bauer J, Liaci AM, Chandra N, Storm RJ, et al. Triazole linker-based trivalent sialic acid inhibitors of adenovirus type 37 infection of human corneal epithelial cells. Org Biomol Chem. 2015;13:9194-205.
3. Messer M, Kerry KR. Milk carbohydrates of the echidna and the platypus. Science. 1973;180:201-3.
4. Chai W, Stoll MS, Galustian C, Lawson AM, Feizi T. Neoglycolipid technology: deciphering information content of glycome. Methods Enzymol. 2003; 362:160-95.
