# Supplementary material for: Extended receptor repertoire of an adenovirus associated with human obesity
Source: PLoS Pathog. 2025 Jan 30;21(1):e1012892. doi: 10.1371/journal.ppat.1012892 (PMC11813153; doi:10.1371/journal.ppat.1012892)
Supplement: Attachment — Submitted filename: Review_PPATHOGENS-D-24-02313.pdf [file ppat.1012892.s020.pdf]

The submitted work explores the receptor profile of Ad36, a virus linked to obesity in animals and potentially humans, using structural biology and virology methods. The authors found that the fiber knob domain (FK) is distinctively structured, allowing it to bind with multiple receptors, including CAR and sialic acid-containing glycans. Ad36 shows a preference for an uncommon sialic acid variant, 4-O,5-N-diacetylneuraminic acid, which has been observed in animals but not humans. These findings suggest Ad36 has adapted to a unique receptor set, likely influencing its host specificity and pathogenic profile. This is a substantial body of data and work that could immediately impact Ad studies by revealing new targets for understanding the role of Ad36 in obesity - as well as its potential cross-species transmission pathways.

The entire manuscript and the supporting information, including all figures, need to be thoroughly reviewed and revised. Here are some comments and suggestions (major and minor) for the authors to include in a revised version:

1. Introduction and study design

- Lines 96-111: Refocus the introduction on the main study objective: adenovirus receptor binding. Currently, the emphasis on the D36 and obesity controversy is off-topic. Expand on receptor interactions and reduce unrelated content.
- Lines 70-72: Expand on receptor interactions, which are currently only briefly mentioned.
- Lines 91-94: Place Ad2 receptor binding into a more relevant context.
- Line 193: The phrase "as expected" is unclear because Factor X was not previously mentioned. Either introduce Factor X earlier or explain why this result is expected.
- Line 207: Correct "typ" to "type" and clarify whether this applies to all D types or only D37-like types.
- Lines 207-208: Add missing references to substantiate statements about the D types.
- Clarify why A549 cells were compared with adipose cell lines instead of using an alternative like 293 cells. Explain the reasoning for this choice to strengthen the experimental design.

2. Further experimental validation

- Consider validating virus entry using authentic cellular models, such as animal cells expressing 4-O,5-N-diacetylneuraminic acid. This approach would offer more relevant insights into Ad36 receptor engagement in natural cellular environments.

3. Statistical analysis and data presentation

- Add a "Statistical analyses" section to the manuscript and specify the statistical tests used. Additionally, include the names of these tests in the figure legends.
- Lines 131-132: Clarify the significance of Bromelain's effects at 20 mU compared to 0 mU. Ensure non-significant results are consistently indicated, as panels B and C lack these indicators, whereas D, E, and F do.
- Lines 154-156: Please report IC50 values only when curves are complete, with at least two points at 100% and 0%. The Ad36 curve does not reach 0%, and the IC50 appears to be over 30 mM, not 25 mM, based on the graph.
- Line 190f: The data could be provided in the SI.

4. Figures and figure captions

- References to figures differ between the manuscript and the supporting information (SI). Standardize the formatting—currently, the manuscript uses parentheses, while the SI uses bold text without parentheses.
- Figure 1C: The y-axis label reads "Geo Mean," while the caption describes it as "knob binding to cells, represented as CPM." Ensure these are aligned and accurate.
- Figure 2A to D: When showing "untreated" versus "treated" comparisons, clarify what control was subtracted or how it was factored into the analysis.
- Figure 4:
    - The caption style differs from Figures 1-3 and includes results. Make style of captions consistent across all figures.
    - Clarify what the error bars in Figure 4B represent (e.g., standard deviation, standard error, etc.).
- Figures 5E and 5F: Review the labels for accuracy and alignment, as panel D also appears slightly misaligned.
- Figure S1:
    - Ensure the cell line "HEP-G2" is presented consistently, either with or without a hyphen.
    - Define "CPM" in the caption.
    - Verify whether the differences in binding shown in Figure S1B are statistically significant.
- Figure S2:
    - Correct the inconsistency in the description of experiments: the caption says "three times with duplicate samples," while the SI mentions "two individual experiments with duplicate samples." Align these details.
    - Virus names in Figure S2G should match those in the main text.
    - In Figure S2H, ensure both Ad36 and Ad5 are correctly mentioned, since C5 was tested but only Ad36 is noted.
- Figure S4: The caption style differs from the rest, mentioning results. Make it consistent.
- Figure S4C: Replace "however" with a more suitable transition, as the sentence does not present a contrast.
- Figure S5:
    - S5A: Specify the color for the DG loop and capitalize "HAdV" correctly.
    - S5C: Include the missing color reference for the YGT trimer.
    - S5D: Prefer the word "asterisk(s)" over symbols ("*") in captions and simplify overly detailed descriptions.

- Figure S6: Consider relocating some details to the main text, especially the note about D15 not being assigned.

5. Supporting information

- Page 3: Correct "decrisbed" to "described" and ensure uniform spelling of "Ad36," including the correct use of hyphens and "D."
- Cell-binding assays: The SI lacks references to some reagents like tunicamycin and benzylGalNAc (used in Figures S2D and 1). List all reagents or explicitly state that various enzymes were used, as described in the figures.
- Cells and viruses paragraph: Include information on Ad12 and other details shown in the SI, which are currently missing from this section.

6. Technical and formatting errors

- Line 139: Correct the lowercase "d" in "D37."
- Line 174 and elsewhere: Ensure "RMSD" is consistently written in uppercase.
- Line 183: The temperature listed as 64.6°C should match the data in Figure S3, which states 62.4°C at pH 2-3. Reconcile this discrepancy.
- Lines 207-208: Specify if the observation applies to all D types or is limited to D37-like types.
- Lines 233-235: Rephrase the description of Ad36's dependency on Neu5Ac, as the current wording is confusing. Emphasize that Ad37 is more affected due to stronger binding to Neu5Ac.
- Lines 391-394: It would be beneficial to test if other D types similar to Ad36 also bind Neu4,5Ac2.
- Line 473: Standardize the spelling of virus names, ensuring consistency in the use of hyphens and capitalization.
- Lines 467-490: Ensure all superscript numbers are properly formatted.
- Line 821: Note that a reduction is also observed in Ad37.
- Line 826: Use consistent formatting for units: "ug/mL" should have a lowercase "L," as used in the figures (or vice versa).
