# Supplementary material for: Extended receptor repertoire of an adenovirus associated with human obesity
Source: PLoS Pathog. 2025 Jan 30;21(1):e1012892. doi: 10.1371/journal.ppat.1012892 (PMC11813153; doi:10.1371/journal.ppat.1012892)
Supplement: Attachment — Submitted filename: Rebuttal reviewer 1 and 2.pdf [file ppat.1012892.s021.pdf]

Reviewer #1 comments:

The submitted work explores the receptor profile of Ad36, a virus linked to obesity in animals and potentially humans, using structural biology and virology methods. The authors found that the fiber-knob domain (FK) is distinctively structured, allowing it to bind with multiple receptors, including CAR and sialic acid-containing glycans. Ad36 shows a preference for an uncommon sialic acid variant, 4-O,5-N-diacetylneuraminic acid, which has been observed in animals but not humans. These findings suggest Ad36 has adapted to a unique receptor set, likely influencing its host specificity and pathogenic profile. This is a substantial body of data and work that could immediately impact Ad studies by revealing new targets for understanding the role of Ad36 in obesity - as well as its potential cross-species transmission pathways.

The entire manuscript and the supporting information, including all figures, need to be thoroughly reviewed and revised. Here are some comments and suggestions (major and minor) for the authors to include in a revised version:

1. Introduction and study design

• Lines 96-111: Refocus the introduction on the main study objective: adenovirus receptor binding. Currently, the emphasis on the D36 and obesity controversy is off-topic. Expand on receptor interactions and reduce unrelated content.

- Response: We agree, we changed the introduction accordingly (see next two points also).

• Lines 70-72: Expand on receptor interactions, which are currently only briefly mentioned.

- Response: Updated as suggested. See lines 80-98.

• Lines 91-94: Place Ad2 receptor binding into a more relevant context.

- Response: We chose to remove this part from the main text.

• Line 193: The phrase "as expected" is unclear because Factor X was not previously mentioned. Either introduce Factor X earlier or explain why this result is expected.

- Response: Thanks. We have now explained the expected binding of Factor X to HAdV-C5 between lines 91-94 in the revised manuscript (track changes version).

• Line 207: Correct "typ" to "type" and clarify whether this applies to all D types or only D37-like types.

- Response: Thanks. Typ corrected to type. This statement applies to multiple HAdV-D types including D37 type. We have re-arranged the lines to make it clear. See lines 230-245 in the revised manuscript (track changes version).

• Lines 207-208: Add missing references to substantiate statements about the D types.

- Response: Several references now added. See line 238 in revised manuscript (track changes version).

• Clarify why A549 cells were compared with adipose cell lines instead of using an alternative like 293 cells. Explain the reasoning for this choice to strengthen the experimental design.

- Response: We chose to compare A549 cells with the adipose cells since in our hands most human AdVs bind to and infect/replicate very well in A549 cells.

2. Further experimental validation

• Consider validating virus entry using authentic cellular models, such as animal cells expressing 4-O,5-N-diacetylneuraminic acid. This approach would offer more relevant insights into Ad36 receptor engagement in natural cellular environments.

- Response: This is a good suggestion, and we actually tried with such cells that according to literature would express 4-O-5-N-diacetylneuraminic acid, but the lectins we received to validate the expression did not work in our hands. Thus, we did not know if we actually had 4-O,5-N diacetylneuraminic acid-expressing cells or not. If/when we get lectins that works, we will do this. Currently, we consider this not to be within the scope of the current manuscript and we hope that reviewer 1 will agree.

3. Statistical analysis and data presentation

• Add a "Statistical analyses" section to the manuscript and specify the statistical tests used. Additionally, include the names of these tests in the figure legends.

- Response: Good suggestion. A separate section "Statistical analyses" has been added in the Materials and Methods (lines 628-633) and all the figure legends have been updated accordingly.

• Lines 131-132: Clarify the significance of Bromelain's effects at 20 mU compared to 0 mU. Ensure non-significant results are consistently indicated, as panels B and C lack these indicators, whereas D, E, and F do.

- Response: There was no statistical significance between 0 and 20 mU Bromelain (now indicated in new figure Figure S2B. We have also doublechecked all data in figure S2B and C and confirm that there is no statistically significant changes. We marked with asterisks where there is a statistical significance and when judged needed, we indicated with ns (for no significance).

• Lines 154-156: Please report IC50 values only when curves are complete, with at least two points at 100% and 0%. The Ad36 curve does not reach 0%, and the IC50 appears to be over 30 mM, not 25 mM, based on the graph.

- Response: We deleted the part of the sentence that described IC50 values.

• Line 190f: The data could be provided in the SI.

- Response: I agree, we have updated the Figure S4 and the text in the manuscript (see lines 219-222).

4. Figures and figure captions

• References to figures differ between the manuscript and the supporting information (SI). Standardize the formatting—currently, the manuscript uses parentheses, while the SI uses bold text without parentheses.

- Response: Thanks, now corrected.

• Figure 1C: The y-axis label reads "Geo Mean," while the caption describes it as "knob binding to cells, represented as CPM." Ensure these are aligned and accurate.

- Response: Corrected, see lines 941-947.

• Figure 2A to D: When showing "untreated" versus "treated" comparisons, clarify what control was subtracted or how it was factored into the analysis.

- Response: The figure legend has been corrected according to the suggestion. Untreated cells or absence of compounds were used as controls. In Figure 2A to D, we plotted virion binding to cells as % of control.

• Figure 4:

o The caption style differs from Figures 1-3 and includes results. Make style of captions consistent across all figures.

- Response: Style of captions is now consistent across all figures.

o Clarify what the error bars in Figure 4B represent (e.g., standard deviation, standard error, etc.).

- Response: The error bars in Figure 4B represent half of the difference between the two values. This was only mentioned in Materials and Methods but now also in figure legend.

• Figures 5E and 5F: Review the labels for accuracy and alignment, as panel D also appears slightly misaligned.

- Response: Corrected as suggested.

• Figure S1:

o Ensure the cell line "HEP-G2" is presented consistently, either with or without a hyphen.

- Response: Corrected as suggested.

o Define "CPM" in the caption.

- Response: CPM defined in caption.

o Verify whether the differences in binding shown in Figure S1B are statistically significant.

- Response: Statistical significance verified and now also shown in figure S1B.

• Figure S2:

o Correct the inconsistency in the description of experiments: the caption says "three times with duplicate samples," while the SI mentions "two individual experiments with duplicate samples." Align these details.

- Response: Corrected in SI text. It is indeed three times with duplicate samples.

o Virus names in Figure S2G should match those in the main text.

- Response: Figure S2G updated to match text.

o In Figure S2H, ensure both Ad36 and Ad5 are correctly mentioned, since C5 was tested but only Ad36 is noted.

- Response: HAdV-C5 now mentioned also in Figure S2 legend.

• Figure S4: The caption style differs from the rest, mentioning results. Make it consistent.

- Response: Made the corrections suggested. See the Figure S4 caption.

• Figure S4C: Replace "however" with a more suitable transition, as the sentence does not present a contrast.

- Response: Corrected.

• Figure S5:

o S5A: Specify the color for the DG loop and capitalize "HAdV" correctly.

- Response: Updated as suggested.

o S5C: Include the missing color reference for the YGT trimer.

- Response: Updated as suggested.

o S5D: Prefer the word "asterisk(s)" over symbols ("*") in captions and simplify overly detailed descriptions.

- Response: Corrected as suggested.

• Figure S6: Consider relocating some details to the main text, especially the note about D15 not being assigned.

- Response: updated the figure legend as suggested.

5. Supporting information

• Page 3: Correct "decrisbed" to "described" and ensure uniform spelling of "Ad36," including the correct use of hyphens and "D."

- Response: Corrected as suggested.

• Cell-binding assays: The SI lacks references to some reagents like tunicamycin and

benzylGalNAc (used in Figures S2D and 1). List all reagents or explicitly state that various enzymes were used, as described in the figures.

- Response: Updated as suggested.

• Cells and viruses paragraph: Include information on Ad12 and other details shown in the SI, which are currently missing from this section.

- Response: updated the ''cells and viruses'' section in Materials and Methods

6. Technical and formatting errors

• Line 139: Correct the lowercase "d" in "D37."

- Response: Corrected as suggested. See line 169

• Line 174 and elsewhere: Ensure "RMSD" is consistently written in uppercase.

- Response: Corrected as suggested. See lines 204, 205

• Line 183: The temperature listed as 64.6°C should match the data in Figure S3, which states 62.4°C at pH 2-3. Reconcile this discrepancy.

- Response: Corrected as suggested.

• Lines 207-208: Specify if the observation applies to all D types or is limited to D37-like types.

- Response: The statement, made in lines 207-208 - Several other HAdV-D types have been reported to use CD46 as a cellular receptor, applies to multiple HAdV-D types including D37 type. I have re-arranged the lines in order to make it clearer. See the changes made between lines 230-245.

• Lines 233-235: Rephrase the description of Ad36's dependency on Neu5Ac, as the current wording is confusing. Emphasize that Ad37 is more affected due to stronger binding to Neu5Ac.

- Response: Rephrased and emphasized as suggested. See lines 270-273.

• Lines 391-394: It would be beneficial to test if other D types similar to Ad36 also bind Neu4,5Ac2.

- Response. We agree with reviewer 1 in that this would be interesting, but we chose to focus on HAdV-D36 as this human AdV type appears unique given its proposed pathogenicity (obesity) and broad species specificity. We also feel that the manuscript is already quite massive. Nevertheless, we will consider this in future studies.

• Line 473: Standardize the spelling of virus names, ensuring consistency in the use of hyphens and capitalization.

- Response: Corrected as suggested.

• Lines 467-490: Ensure all superscript numbers are properly formatted.

- Response: Corrected as suggested.

• Line 821: Note that a reduction is also observed in Ad37.

- Response: Figure 2 legend has been updated.

• Line 826: Use consistent formatting for units: "ug/mL" should have a lowercase "L," as used in the figures (or vice versa).

- Response: Corrected as suggested.

Reviewers' Comments:

Reviewer's Responses to Questions

Part I - Summary

Please use this section to discuss strengths/weaknesses of study, novelty/significance, general execution and scholarship.

Reviewer #1: See attached pdf.

Reviewer #2: Manuscript entitled "Extended receptor repertoire of an adenovirus associated with human obesity" by Liaci et al describes the receptor usage of HAdV-D36, which is implicated in the obesity in animals and humans. The manuscript comes from the highly reputed research laboratories in virology and structural biology and reports the results on an important topic of adenoviral entry mechanisms and is well written. Therefore, it is well suited for publication in PLOS Pathogens.

I have a few comments on the scope of the findings and minor suggestions.

1) Authors say in the abstract that the elongated DG loop of the Ad36 fiber knob (FK) alters the known CAR binding interfaces and "heavily clashes" with CAR when superimposed onto a known FK-CAR complex structure (Fig. S4). These statements imply that Ad36-FK is unlikely to bind to CAR. But authors show using cell-based assays that the presence of CAR increases the binding of Ad36 in some of the cell lines tested (Fig. 1C). Is it possible that Ad36 actually binds to sugar moieties (e.g., sialic acids) on glycosylated CAR? Is there a way to prove or disprove such a possibility (e.g., deglycosylation of CAR expressed on CHO cells)?

- Response: The statement in the abstract that ''the elongated DG loop of the Ad36 fiber knob (FK) alters the known CAR binding interfaces for established adenovirus receptors'' is made based on our cell-based assay and structural data. Structural data (Figure S4) shows that the canonical CAR binding domain clashes with the long DG loopAs, however we also show that the HAdV-D36 FK readily forms a stable complex with the distal extracellular domain of CAR (CAR-D1) in vitro (Figure S4C). Therefore, the observed clash between the DG loop of the counter clockwise adjacent FK monomer and CAR should be overcome, and that could only be possible by a major rearrangement in the loop involving the dissociation of the inter-monomer contacts, or by altering the binding region.

Yes, it is possible that CAR can be glycosylated. There are two glycosylation sites; one at N106 D1-domain, and another at N206 in D2-domain. Since we showed that inhibiting N-glycosylation by treating cells with tunicamycin causes non-significant reduction of the virus binding (Figure S2D), we do not think that HAdV-D36 binds to sialylated-CAR.

There is another way to prove or disprove such possibility is to mutate the respective amino acid and then express the mutated CAR on CHO cells and compare the binding (Sandra Pinkert et.al., J Virol. 2016 May 27;90(12):5601–5610. doi: 10.1128/JVI.00315-16). However, we think that this is beyond the scope of the current study, which is already quite massive (in our opinion).

2) Is there a requirement that Neu5Ac moieties to be the terminal sugars as part of the complex carbohydrates to bind to Ad-FKs. Authors may be able to answer this question from the known organization of sugars from the glycan array they used in the study. Along those lines, can more than two consecutive sugars (as part of the complex carbohydrates) bind to the 2-3 binding sites on a FK?

- Response: Different Ad-FKs may have varying preferences and modes for sialic acid binding. Internal Neu5Ac residues are known to contribute to FK binding, such as the terminal and internally branched 2-3-linked Neu5Ac residues in GD1a glycan simultaneously bound by the trimeric Ad37 FK (doi: 10.1038/nm.2267), as well as the internal 2-8-linked Neu5Ac residues, in addition to the terminal NeuAc in the polysialic acid sequence bound by the Ad52 short FK (doi: 10.1073/pnas.1716900115). Both papers contain glycan array data. In the present study of Ad36-FK, as discussed in the manuscript, the lack of diverse glycan probes carrying 4-O-Ac Neu5Ac could explain the highly restricted binding observed with Ad36 FK.

3) Figure 3. Since the authors are comparing the relative electrostatic potentials of Ad36 and Ad37 FKs to make a point, it would be useful to include a panel showing the electrostatic potential of Ad37-FK in addition to that of Ad36-FK. Moreover, in the reviewer's opinion, the superposition of FKs (Panel B) may be visualized better as a ribbon representation than as a surface representation.

- Response: We thank reviewer 2 for this suggestion. We have updated the Figure 3 now. We now include an ESP surface presentation of HAdV-D37FK for direct comparison. We tested various options for the visualization. We now include a surface view with increased transparency of the HAdV-D36FK and a ribbon representation of the superposed beta sheets. We believe that together these two views should convey the point effectively.

Minor comment.

Line 33 (Page 2). In the abstract…HAdV-D36 has been causally linked…

consider replacing "causally" with "putatively"

- Response: Updated as suggested.

Part II – Major Issues: Key Experiments Required for Acceptance

Please use this section to detail the key new experiments or modifications of existing experiments that should be absolutely required to validate study conclusions.

Generally, there should be no more than 3 such required experiments or major modifications for a "Major Revision" recommendation. If more than 3 experiments are necessary to validate the study conclusions, then you are encouraged to recommend "Reject".

Reviewer #1: See attached pdf.

Reviewer #2: (No Response)

Part III – Minor Issues: Editorial and Data Presentation Modifications

Please use this section for editorial suggestions as well as relatively minor modifications of existing data that would enhance clarity.

Reviewer #1: See attached pdf.

Reviewer #2: (No Response)

____

PLOS authors have the option to publish the peer review history of their article (what does this mean?). If published, this will include your full peer review and any attached files.

If you choose “no”, your identity will remain anonymous but your review may still be made public.

Do you want your identity to be public for this peer review? For information about this choice, including consent withdrawal, please see our Privacy Policy.

Reviewer #1: No

Reviewer #2: Yes: Vijay S. Reddy

[NOTE: If reviewer comments were submitted as an attachment file, they will be attached to this email and accessible via the submission site. Please log into your account, locate the manuscript record, and check for the action link "View Attachments". If this link does not appear, there are no attachment files.]
